# Branched-chain α-ketoacids are preferentially reaminated and activate protein synthesis in the heart

**DOI:** 10.1038/s41467-021-21962-2

**Published:** 2021-03-15

**Authors:** Jacquelyn M. Walejko, Bridgette A. Christopher, Scott B. Crown, Guo-Fang Zhang, Adrian Pickar-Oliver, Takeshi Yoneshiro, Matthew W. Foster, Stephani Page, Stephan van Vliet, Olga Ilkayeva, Michael J. Muehlbauer, Matthew W. Carson, Joseph T. Brozinick, Craig D. Hammond, Ruth E. Gimeno, M. Arthur Moseley, Shingo Kajimura, Charles A. Gersbach, Christopher B. Newgard, Phillip J. White, Robert W. McGarrah

**Affiliations:** 1 Duke Molecular Physiology Institute, Duke University School of Medicine, Durham, NC USA; 2 Department of Medicine, Division of Cardiology, Duke University School of Medicine, Durham, NC USA; 3 Department of Medicine, Division of Endocrinology, Metabolism and Nutrition, Duke University School of Medicine, Durham, NC USA; 4 Sarah W. Stedman Nutrition and Metabolism Center, Duke University School of Medicine, Durham, NC USA; 5 Department of Biomedical Engineering, Duke University, Durham, NC USA; 6 Center for Advanced Genomic Technologies, Duke University, Durham, NC USA; 7 UCSF Diabetes Center, San Francisco, CA USA; 8 Duke Proteomics and Metabolomics Shared Resource, Duke University School of Medicine, Durham, NC USA; 9 Eli Lilly and Company, Indianapolis, IN US; 10 Department of Surgery, Duke University School of Medicine, Durham, NC USA; 11 Department of Pharmacology and Cancer Biology, Duke University, Durham, NC USA

**Keywords:** Metabolomics, Cardiac hypertrophy, Metabolism

## Abstract

Branched-chain amino acids (BCAA) and their cognate α-ketoacids (BCKA) are elevated in an array of cardiometabolic diseases. Here we demonstrate that the major metabolic fate of uniformly-^13^C-labeled α-ketoisovalerate ([U-^13^C]KIV) in the heart is reamination to valine. Activation of cardiac branched-chain α-ketoacid dehydrogenase (BCKDH) by treatment with the BCKDH kinase inhibitor, BT2, does not impede the strong flux of [U-^13^C]KIV to valine. Sequestration of BCAA and BCKA away from mitochondrial oxidation is likely due to low levels of expression of the mitochondrial BCAA transporter SLC25A44 in the heart, as its overexpression significantly lowers accumulation of [^13^C]-labeled valine from [U-^13^C]KIV. Finally, exposure of perfused hearts to levels of BCKA found in obese rats increases phosphorylation of the translational repressor 4E-BP1 as well as multiple proteins in the MEK-ERK pathway, leading to a doubling of total protein synthesis. These data suggest that elevated BCKA levels found in obesity may contribute to pathologic cardiac hypertrophy via chronic activation of protein synthesis.

## Abstract (web summary)

Systemic modulation of branched-chain keto acid (BCKA) metabolism alters cardiac health. Here, the authors define the major fates of BCKA in the heart and demonstrate that acute exposure to BCKA levels found in obesity activates cardiac protein synthesis and markedly alters the heart phosphoproteome.

## Introduction

The association of branched-chain amino acids (BCAA–valine, leucine, isoleucine) and their α-ketoacids (BCKA) with metabolic diseases such as obesity, insulin resistance, and diabetes are now well documented^1–4^, and mechanisms underlying these associations are beginning to emerge^5–8^. The role of BCKA metabolism in cardiac health and disease is less well understood. Recently it was reported that the absence of PPM1K (also called PP2Cm), the phosphatase responsible for dephosphorylation and activation of the branched-chain α-ketoacid dehydrogenase (BCKDH) complex, leads to cardiac contractile dysfunction in zebrafish and mice^9,10^. Impaired cardiac BCAA and BCKA metabolism have also been observed in animal models of pressure-overload-induced heart failure, myocardial infarction-induced heart failure, and myocardial ischemia^11–13^.

Because the foregoing studies involved whole-body knockout of PPM1K and/or administration of a small molecule inhibitor of the BCKDH kinase (BDK), 3,6-dichlorobenzo[b]thiophene-2-carboxylic acid (BT2)^14^, which serves to activate BCKDH in all tissues, the impact of specific alteration of cardiac BCAA metabolism on cardiovascular disease phenotypes remains to be defined. This is especially true given that BCAA metabolism is differentially regulated in various organs and tissues in response to cardiometabolic disease-inducing conditions such as obesity and overnutrition^6,15^.

To address this knowledge gap, we employed stable-isotope tracing in isolated perfused hearts and in living animals to specifically map the fates of BCKA in the heart. Surprisingly, we find that the isolated heart uses only a modest fraction of BCKA for mitochondrial oxidation to the level of tricarboxylic acid (TCA) cycle intermediates. Instead, the major fate of BCKA is conversion to BCAA via “reamination” catalyzed by the branched-chain amino acid transaminase (BCAT) reaction. We applied small molecule inhibitors of BCAT and BDK; AAV9-mediated, cardiac-specific overexpression of the recently identified mitochondrial BCAA carrier, SLC25A44; and global phosphoproteomics analysis to define the mechanisms that regulate BCKA reamination. Notably, we demonstrate that provision of BCKA to the isolated heart at levels found in obesity causes robust stimulation of cardiac protein synthesis in concert with activation of key elements of the protein biosynthetic machinery. These results outline mechanisms by which the chronic elevations of BCKA observed in obese, insulin-resistant states may activate cardiac hypertrophy to contribute to the pathogenesis of cardiovascular diseases.

## Results

### Reamination is the major fate of BCKA in the isolated perfused heart

**Fig. 1:**
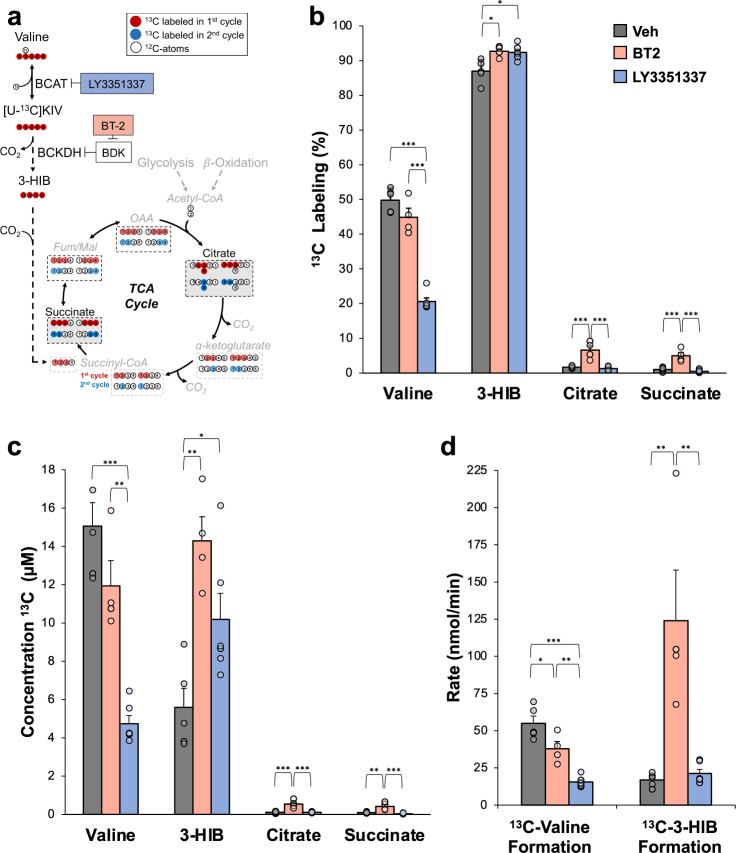
Preferential reamination of α-ketoisovalerate (KIV) to valine in the isolated perfused heart. **a** Simplified schematic figure diagraming the potential metabolic fates of [U-^13^C]KIV, as well as the effects of LY3351337 to inhibit BCAT activity, and BT2 to inhibit BDK. Label incorporation from first (red circles) and second (blue circles) passes through the TCA cycle are shown in dashed boxes. Measured metabolites in the TCA cycle are highlighted with a gray background and black dashed border. **b** The fractional percent labeling with ^13^C is shown for the indicated metabolites; (**c**) The absolute amounts of ^13^C-labeled valine, 3-HIB, citrate, and succinate are shown. Data in **b–d** are from hearts isolated from Wistar rats and perfused with [U-^13^C]KIV (100 μM) in the absence (Veh; *n* = 5; gray) or presence of the BDK inhibitor, BT2 (*n* = 4; red) or the BCAT inhibitor, LY3351337 (*n* = 6; blue). **d** Rate of reamination (nmol/min) of [U-^13^C]KIV to valine and rate of formation of ^13^C-labeled 3-HIB from [U-^13^C]KIV in isolated hearts following treatment with LY3351337 or BT2. Data represent mean ± SEM. Statistical differences indicated by Tukey’s HSD post-hoc test following one-way ANOVA: *P < 0.05, **P < 0.005, ***P < 0.0005.

We set out to map the fate of BCKA using stable isotope tracing in an isolated perfused heart system. Hearts were isolated from lean Wistar rats and perfused in Langendorff mode with physiologic concentrations of palmitate, glucose, amino acids, and uniformly-^13^C-labeled α-ketoisovalerate ([U-^13^C]KIV), the α-ketoacid of valine. We used the α-ketoacid of valine rather than leucine or isoleucine in these studies to allow measurement of the labeling of the valine/KIV-derived metabolite, 3-hydroxyisobutyrate (3-HIB), which has been reported to promote transendothelial fatty acid transport^16^ (Fig. 1a). Labeling patterns of intermediates shown in Fig. 1a are based in part on previous NMR-based measurements in hearts perfused with stable isotope-labeled substrates^17–19^. Consistent with reports from other laboratories^20,21^, we observed that perfusion of hearts with [U-^13^C]KIV caused only modest labeling of the TCA cycle intermediates citrate and succinate (<2%) (Fig. 1b). In contrast, the valine and 3-HIB pools were robustly labeled (49.8 ± 1.5% and 87.0 ± 1.5%, respectively) under these conditions (Fig. 1b). Since the concentration of labeled valine exceeds that of 3-HIB by approximately 3-fold, we conclude that reamination to valine is the major metabolic fate of KIV in the heart (Fig. 1c). To determine if the robust reamination of KIV to valine applies to other BCKA, we also perfused isolated hearts with α-ketoisocaproate labeled in the 1- and 2-carbon positions ([1,2-^13^C]KIC). Similar to our findings with labeled KIV, perfusion of hearts with [1,2-^13^C]KIC resulted in robust labeling of leucine (80.2 ± 4.6%) (Supplementary Fig. 1a).

### Activation of BCKDH in the isolated perfused heart does not alter ^13^C-valine enrichment

We next sought to determine if BCKDH activity is a major determinant of KIV fate in the isolated heart. To this end, we pre-treated Wistar rats with the BDK inhibitor BT2 (20 mg/kg i.p.) for three days to cause global activation of BCKDH, including in the heart. We then performed isolated heart perfusion studies as described above, with the provision of [U-^13^C]KIV as labeled substrate.

Pre-treatment of rats with BT2 caused an approximately 3-fold increase in cardiac BCKDH enzymatic activity relative to hearts from vehicle-treated control rats (Supplementary Fig. 1b), similar to what has been observed in mitochondria isolated from mice pre-treated with BT2^22^. However, this had no effect on KIV reamination to valine measured as percent labeling or total ^13^C-valine concentration in the heart (Fig. 1b and 1c). BT2 administration did cause a 3-fold increase in ^13^C−3-HIB concentration and also resulted in increased labeling of the TCA cycle intermediates citrate and succinate (Fig. 1c).

### BCAT inhibition in the isolated perfused heart blunts KIV reamination but does not increase labeling of TCA cycle intermediates

To determine whether reamination of KIV by the branched-chain transaminase (BCAT) reaction limits access of KIV to other metabolic pathways in the heart, we next perfused isolated hearts with [U-^13^C]KIV in the presence of a BCAT inhibitor, LY3351337^23^, during the perfusion period (40 μM; Eli Lilly Co.). LY3351337 inhibits both the cytoplasmic (BCAT1) and mitochondrial (BCAT2) forms of BCAT but is not active against other common transaminases such as alanine or aspartate transaminases (Supplementary Fig. 2). As expected, LY3351337 potently and significantly (*P* = 1.27 × 10^−5^) lowered labeled valine content in extracted heart tissue by ~70%, while causing an approximate doubling of labeled 3-HIB concentrations compared to vehicle treatment (*p* = 0.04; Fig. 1c). However, LY3351337 had no significant effect on labeled citrate content and actually lowered labeled succinate concentration by ~75%. Given the weak labeling of TCA cycle intermediates, including succinate, a direct catabolic product of valine and KIV metabolism, our data suggest that flux of labeled KIV through its oxidative pathway mostly terminates at 3-HIB in the rat heart. This may be due to the fact that 3-HIB is the only product of valine/KIV catabolism that is not CoA modified after irreversible oxidative decarboxylation by BCKDH, a feature that allows it to cross mitochondrial and cellular membranes, thereby possibly limiting its further metabolism.

To measure the rate of [U-^13^C]KIV reamination to ^13^C-valine in perfused rat hearts, we determined the concentration of ^13^C-labeled valine in the perfusate medium from the isolated heart experiments performed in the presence or absence of treatment with LY3351337 or BT2. Treatment with BT2 lowered the absolute concentration of ^13^C-labeled valine in the heart perfusate medium by about 40%, whereas treatment with LY3351337 reduced labeling by nearly 80% (Supplementary Fig. 1c). By combining the absolute labeling of ^13^C-valine in the heart perfusate (Supplementary Fig. 1c) with that for the heart tissue (Fig. 1c), we derived a total rate of reamination of [U-^13^C]KIV to ^13^C-labeled valine (see “Methods” section for a description of this calculation) under the three experimental conditions. Treatment of rats with BT2 caused an approximate 25% decrease in total reamination rate, whereas a much stronger inhibition of total reamination rate of about 80% was observed in response to BCAT inhibition with LY3351337 (Fig. 1d).

In a similar manner, we derived a total rate of formation of ^13^C-labeled 3-HIB from [U-^13^C]KIV under these three experimental conditions. Consistent with activation of BCKDH (Supplementary Fig. 1b), BT2 treatment increased the concentration of ^13^C-labeled 3-HIB in the heart perfusate medium by approximately 10-fold (Supplementary Fig. 1c) with a corresponding increase in 3-HIB formation rate (Fig. 1d). While BCAT inhibition with LY3351337 increased ^13^C-labeled 3-HIB concentration in the heart tissue (Fig. 1c), there was no significant increase in ^13^C-labeled 3-HIB concentration in the heart perfusate medium (Supplementary Fig. 1c) and, therefore, no significant change in the total rate of ^13^C-labeled 3-HIB formation from [U-^13^C]KIV (Fig. 1d).

Taken together, these experiments show consistently that reamination of KIV to valine and its catabolic conversion to 3-HIB are the main metabolic fates of labeled KIV in the isolated perfused rat heart, while KIV contribution to the TCA cycle is low, even when BCKDH is pre-activated by BT2 treatment. In addition, the strong reamination of KIV to valine is largely not diverted by activation of BCKDH, despite the effect of this maneuver to increase the catabolic flux of KIV to 3-HIB, succinate, and citrate, whereas reamination is strongly suppressed by inhibition of BCAT activity.

### Reamination of KIV to valine is an active pathway in heart in vivo

**Fig. 2:**
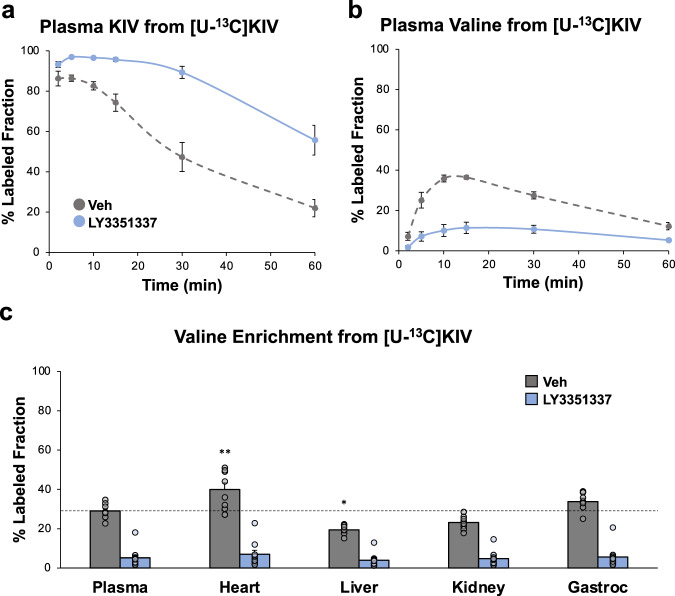
KIV reamination across tissues in living rats. Thirty minutes after a single injection of BCAT inhibitor, LY3351337 (10 mg/kg i.p.; *n* = 5; blue), or Veh (DMSO; *n* = 5; gray), Wistar rats received an i.p. injection of [U-^13^C]KIV (100 mg/kg), followed by blood sampling from the tail vein at 2, 5, 10, 15, 30, and 60 min. Fractional percent labeling with ^13^C of (**a**) plasma KIV and (**b**) plasma valine. *N* = 5–6 per group. A separate cohort of Wistar rats was treated with LY3351337 (10 mg/kg i.p.) or vehicle and received an i.p. injection of [U-^13^C]KIV (100 mg/kg) 30 min later. Tissues were harvested 30 min after the [U-^13^C]KIV injection. **c** Fractional percent labeling with ^13^C of valine in plasma and the indicated tissues in the absence (Veh; *n* = 8) or presence of LY3351337 (*n* = 10). The dashed line indicates the plasma enrichment level. Data represent mean ± SEM. Statistical differences indicated by a two-way paired Student’s *t*-test: *P < 0.05, **P < 0.005.

To determine if our observations of BCKA reamination to BCAA in the isolated heart are reflective of their fate in the heart in vivo, and to more broadly investigate the systemic effects of manipulation of BCAA catabolism, we next studied the fate of [U-^13^C]KIV in Wistar rats. Thirty minutes after a single injection of LY3351337 (10 mg/kg i.p.) or vehicle (DMSO), the rats received an i.p. injection of [U-^13^C]KIV (100 mg/kg), followed by blood sampling from the tail vein at 2, 5, 10, 15, 30, and 60 min. Concomitant with the rapid decline in circulating [U-^13^C]KIV we observed a rapid appearance of labels in the valine pool (Figs. 2a and 2b). Remarkably, 40% of the plasma valine pool was labeled in the first 10 min after [U-^13^C]KIV administration. BCAT inhibition with LY3351337 resulted in a clear delay in clearance of labeled KIV from plasma, with a concomitant decrease in the appearance of labeled valine over the 60-min time course (Figs. 2a and 2b). To determine the relative role of the heart in the reamination of BCKA, we examined ^13^C-valine enrichment in several key metabolic tissues and organs. In these studies, rats were pre-treated with either vehicle or LY3351337 (10 mg/kg i.p.) and received an i.p. injection of [U-^13^C]KIV (100 mg/kg) 30 min later. Animals were sacrificed and tissues harvested 30 min after injection of [U-^13^C]KIV. In control vehicle-treated rats, percent labeling of valine from [U-^13^C]KIV reached approximately 30% in plasma. Across tissues, the labeling of valine from [U-^13^C]KIV was significantly higher in the heart than in plasma (*P* < 0.01, Fig. 2c), but was similar to plasma levels in skeletal muscle and clearly lower in liver and kidney (Fig. 2c). These data suggest that the reamination of BCKA to BCAA is particularly active in the heart. As expected, treatment of the rats with LY3351337 decreased label enrichment in the valine pool of all tissues (Fig. 2c). Given that BCAT activity is known to be very low in the liver and kidney and relatively high in skeletal muscle and heart^24^, these data are consistent with a model in which the heart plays a key role in the reamination of BCKA to BCAA for the entire organism.

### Low levels of the mitochondrial BCAA transporter, SLC25A44, promote accumulation of reaminated BCAA in the heart

**Fig. 3:**
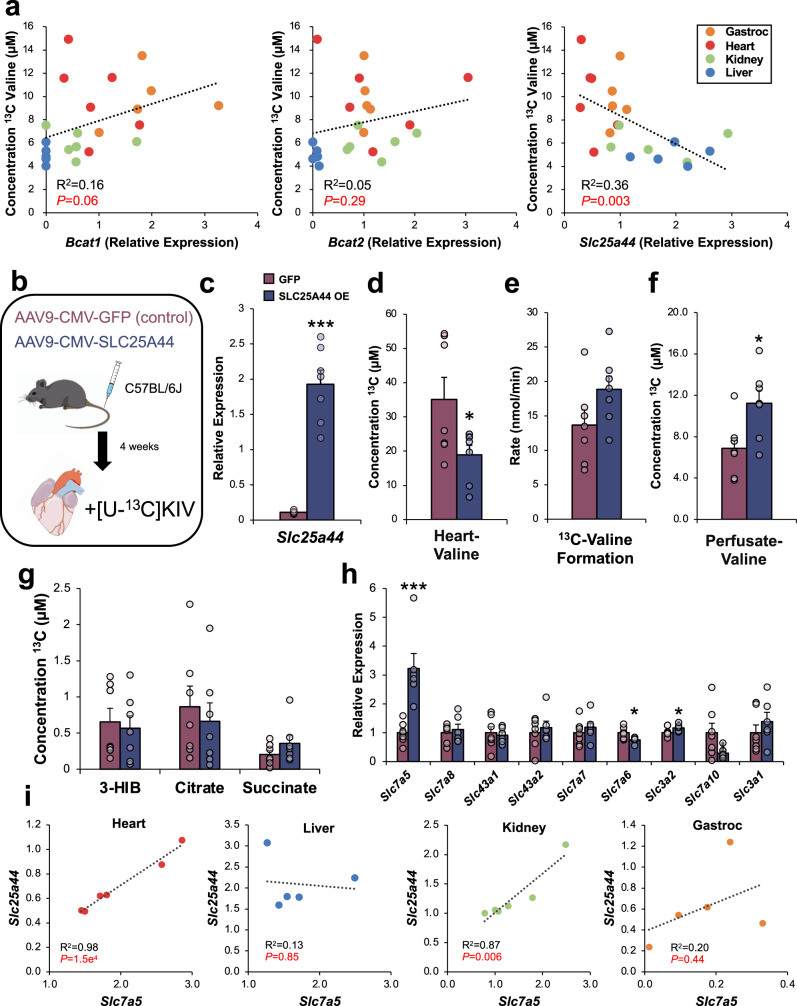
Over-expression of SLC25A44 decreases KIV reamination in the isolated perfused mouse heart. **a** Correlation of ^13^C-valine concentration with *Bcat1*, *Bcat2,* and *Slc25a44* mRNA levels across rat heart (*n* = 6; red), liver (*n* = 5; blue), kidney (*n* = 6; green), and gastrocnemius (*n* = 5; orange). Data for tissue labeling of ^13^C valine was obtained in rats injected with [U-^13^C] KIV, as summarized in Fig. 2C. **b** Experimental design for the study of AAV-mediated overexpression of SLC25A44 in mouse heart. **c** Relative mRNA expression of *Slc25a44* in isolated perfused hearts following treatment with AAV9-CMV-SLC25A44 (*n* = 7; purple) versus AAV9-CMV-GFP (*n* = 7; navy). The concentration of ^13^C-labeled valine in the heart (**d**), the total rate of formation of ^13^C-labeled valine (**e**), perfusate concentration of ^13^C-labeled valine (**f**), and concentrations of ^13^C-labeled TCA cycle intermediates from [U-^13^C]KIV (100 μM) (**g**) following treatment with AAV9-CMV-SLC25A44 or AAV9-CMV-GFP. **h** Relative levels of transcripts encoding BCAA transporters in AAV9-CMV-SLC25A44 versus AAV9-CMV-GFP-treated hearts *N* = 7 per group. **i** Correlations of *Scl25a44* with *Slc7a5* mRNA levels in rat heart, liver, kidney, or gastrocneminus (gastroc) muscle. *N* = 5–6 per group. Data represent mean ± SEM. Statistical differences indicated by Pearson correlation (**a, i**) or a two-way, paired Student’s *t*-test (**c–h**): *P < 0.05, ***P < 0.0005.

Our finding that the heart has the highest valine label enrichment from [U-^13^C]KIV among tissues in living rats is consistent with our results in the isolated perfused heart and suggests that the heart actively reaminates KIV in vivo (Fig. 2c). Furthermore, the lack of effect of the BCKDH activator BT2 on KIV reamination in the perfused heart (Fig. 1c) suggests that upon entry into the cardiomyocytes, a large portion of KIV is rapidly reaminated to valine in the cytosol, sequestering it from metabolism by the mitochondrial BCKDH enzyme complex. To gain further insight into the factors that might explain this cardiac-selective BCKA utilization, we measured mRNA expression of the BCAT isoforms, *Bcat1* (cytosolic) and *Bcat2* (mitochondrial), as well as the newly identified mitochondrial BCAA transporter, *Slc25a44*^25^, normalized to *Rplp0* in heart, liver, kidney and skeletal muscle and examined their relationship to the concentration of labeled valine after injection of [U-^13^C]KIV (Fig. 3a; Supplementary Fig. 3). Consistent with previous reports^24^, *Bcat2* mRNA levels were higher in all tissues surveyed than those for *Bcat1* (Supplementary Fig. 3c). *Bcat2* expression had a tissue expression pattern of heart>kidney>skeletal muscle»liver, whereas the relative abundance of *Bcat1* transcripts had the pattern of skeletal muscle>heart>kidney»liver. However, across these tissues, there was no significant correlation of the extent of valine labeling from [U-^13^C]KIV with either *Bcat1* (*P* = 0.06) or *Bcat2* (*P* = 0.29) expression (Fig. 3a). In contrast, there was a strong inverse correlation between valine labeling and mitochondrial BCAA carrier *Slc25a44* mRNA levels (*R*^2^ = 0.36, *P* < 0.003). This finding suggests that BCAA are preferentially retained in the cytosolic compartment in the heart, thus favoring the accumulation of products of BCKA reamination.

To test whether the low intrinsic expression of cardiac SLC25A44 contributes to the accumulation of BCAA formed by reamination of BCKA in the heart, we expressed SLC25A44 or GFP under control of the CMV promoter in the well-described cardiotropic adeno-associated virus serotype 9 vector^26^. We then administered the SLC25A44 or GFP control AAV9 vectors to mice via tail vein injection, and 4 weeks later, performed [U-^13^C]KIV perfusion studies in hearts isolated from both groups of mice (Fig. 3b). Treatment with AAV9-CMV-SLC25A44 led to a strong induction of *Slc25a44* mRNA expression in the heart compared to hearts from mice treated with AAV9-CMV-GFP control (Fig. 3c). SLC25A44 overexpression was associated with a 50% reduction in the ^13^C-labeled valine pool in the heart (Fig. 3d) without a corresponding change in the overall rate of ^13^C-valine formation (Fig. 3e) due to increased export of ^13^C-valine into the perfusate (Fig. 3f). Despite its described function as a mitochondrial BCAA transporter^25^, SLC25A44 overexpression did not lead to increased ^13^C enrichment in downstream mitochondrial oxidative intermediates of [U-^13^C]KIV (Fig. 3g; Supplementary Fig. 4).

Given that cardiac SLC25A44 overexpression did not impact [U-^13^C]KIV mitochondrial oxidation but increased export of ^13^C-valine into the isolated heart perfusate, we measured the expression of known BCAA plasma membrane transporters to determine if SLC25A44 affected cellular BCAA export. Indeed, isolated hearts with overexpression of SLC25A44 had a three-fold increase in expression of the plasma membrane BCAA transporter *Slc7a5* (also known as *Lat1*) (Fig. 3h). To determine if the correlation of *Slc25a44* and *Slc7a5* expression persists in the absence of SLC25A44 overexpression and whether this relationship exists in other tissues, we measured the expression of these two transporters across tissues of Wistar rats. *Slc25a44* was strongly correlated with *Slc7a5* expression in the heart (*R*^2^ = 0.98, *P* = 1.5e^−4^) and, to a lesser degree, in the kidney (*R*^2^ = 0.87, *P* = 0.006). There was no significant correlation in the liver or gastrocnemius muscle (Fig. 3i). Taken together, these findings highlight a previously unappreciated tissue-specific coordination of plasma membrane and mitochondrial BCAA transport in a subset of tissues, especially the heart.

### BCKA reamination activates protein synthesis in isolated perfused hearts

**Fig. 4:**
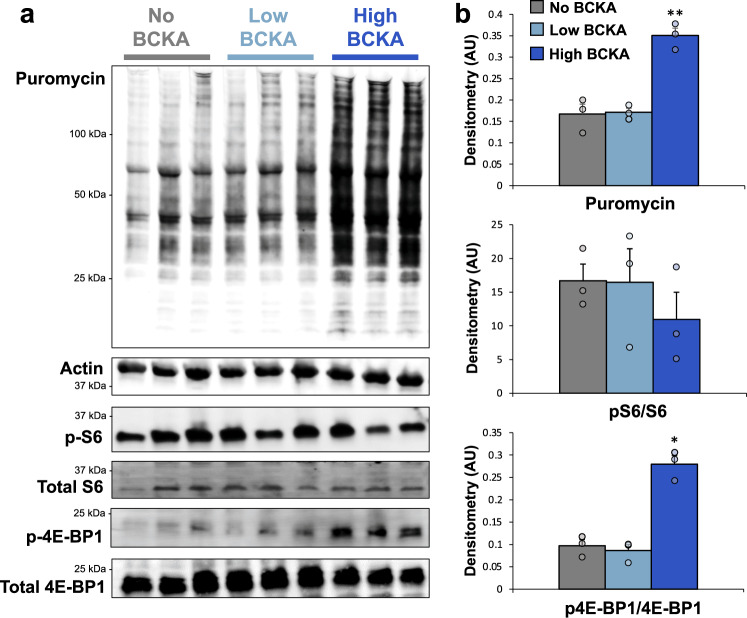
High concentrations of BCKA activate protein synthesis in isolated perfused hearts in concert with increased 4E-BP1 phosphorylation. Wistar rat hearts were isolated and perfused with buffers containing no BCKA (*n* = 3; gray), BCKA at concentrations found in the plasma of lean rats (*n* = 3; light blue) or BCKA at concentrations found in the plasma of obese rats (*n* = 3; dark blue). **a** Upper panel–immunoblot analysis of the incorporation of puromycin into newly synthesized proteins. Lower panel—immunoblot of total and phosphorylated ribosomal protein S6 and 4E-BP1. **b** Densitometric quantification of puromycin labeling (normalized to actin loading control), phosphorylated ribosomal protein S6 (normalized to total S6), and phosphorylated 4E-BP1 (normalized to total 4E-BP1). Data represent mean ± SEM. Statistical differences indicated by Tukey’s HSD post-hoc test following one-way ANOVA: *P < 0.05, **P < 0.005.

The role of BCAA in modulating protein synthesis via activation of mTOR and translation initiation factors has been well-described^27^. Given our finding of robust reamination of BCKA to BCAA in the heart, we hypothesized that BCKA exposure could increase rates of protein synthesis. To test this hypothesis, we used the surface sensing of translation (SUnSET) technique, which measures the incorporation of puromycin (a structural analog of tyrosyl-tRNA) into newly synthesized proteins^28^. We isolated hearts from Wistar rats and perfused them with puromycin (1 μM) and physiologic concentrations of palmitate, glucose, and amino acids, in the absence or presence of concentrations of BCKA matching those found in either Zucker obese or Zucker lean rats^5^. Importantly, levels of BCKA found in Zucker obese rats are comparable to those found in the circulation of animals in the setting of heart failure^11^. Hearts perfused with BCKA at concentrations found in obese rats exhibited clear increases in the incorporation of puromycin into newly synthesized proteins relative to hearts perfused with no BCKA or BCKA concentrations found in lean animals (Fig. 4a). Thus, the strong flux of BCKA to BCAA in the heart, coupled with the chronic increase in BCKA supply as found in obesity^5^ or heart failure^11^ is sufficient to drive an increase in total protein synthesis rates in the heart.

To investigate the mechanisms by which BCKA stimulates protein translation, we examined the phosphorylation state of ribosomal protein S6 (S6) and eukaryotic translation initiation factor 4E-binding protein 1 (4E-BP1). Phosphorylation of either protein serves to increase total protein translation. Exposure of perfused hearts to the high concentration of BCKA found in obese rats had no effect on S6 phosphorylation above the robust levels already observed in hearts perfused with normal or no BCKA (Fig. 4b). In contrast, exposure of hearts to the high concentration of BCKA caused a significant increase in phosphorylation of 4E-BP1 relative to either control group (*P* < 0.05, Fig. 4b), which serves to activate protein synthesis by inactivating the translation repressor function of this protein. These data indicate that acute exposure of the heart to BCKA levels observed in circulation of obese animals is sufficient to promote phosphorylation of 4E-BP1 and activate protein synthesis.

### Impact of acute BCKA exposure on the cardiac phosphoproteome

To gain a broader, unbiased view of the potential mechanisms involved in the cardiac response to elevated BCKA exposure, we next surveyed the phosphoproteomic landscape in hearts that were perfused with BCKA at levels found in obese and lean rats, using isobaric tandem mass tags (TMT) labeling, phosphopeptide enrichment, high pH-reversed phase fractionation, and finally, one-dimensional liquid chromatography-tandem mass spectrometry (1D-LC-MS/MS) analysis of unenriched and phosphopeptide-enriched proteomes. Using this approach we quantified 9057 phosphopeptides from 4724 phosphoproteins (Supplementary Data 1).

**Fig. 5:**
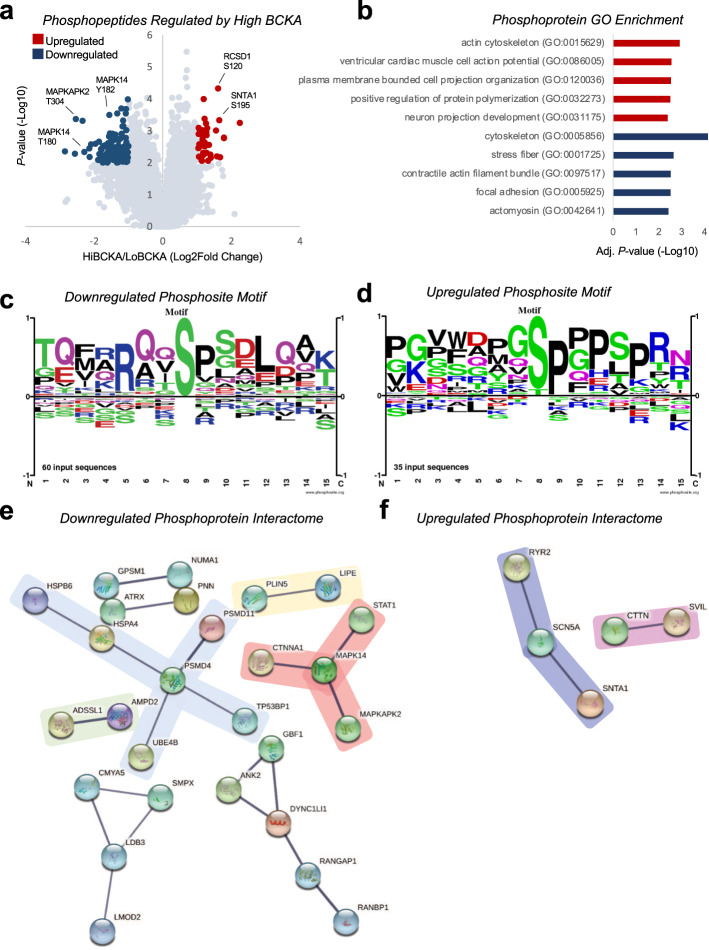
Effect of BCKA exposure on the cardiac phosphoproteome. **a** Volcano plot depicting the 9057 phosphopeptides detected using isobaric tandem mass tags (TMT) labeling and one-dimensional liquid chromatography, tandem mass spectrometry (1D-LC-MS/MS) in hearts perfused with levels of BCKA found in lean (LoBCKA; *n* = 4) or obese (HiBCKA; *n* = 5) rats. Each data point represents the −Log10 *P*-value (*y*-axis) and Log2 fold change HiBCKA/LoBCKA (*x*-axis) for each phosphopeptide following a two-way, paired Student’s t-test. A significance threshold of >2 fold change and *P*-value <0.01 was used. Significantly upregulated phosphopeptides are colored red, significantly downregulated phosphopeptides are colored blue. Phosphopeptides that did not meet the significance threshold are colored gray. **b** The top five overrepresented gene ontology (GO) terms for the significantly upregulated (red) and downregulated (blue) phosphopeptides are shown. Consensus phosphosite motif sequences generated for down and upregulated phosphosites are shown in panels **c** and **d**, respectively. **e**–**f** Protein-protein interaction networks generated in STRING v11 for significantly downregulated (**e**) and upregulated (**f**) phosphoproteins.

Employing a significance threshold of 2-fold change and *P*-value <0.01, 39 phosphopeptides from 28 unique phosphoproteins were upregulated and 111 phosphopeptides from 83 unique phosphoproteins were downregulated in response to perfusion of hearts with levels of BCKA found in obese compared to lean animals (Fig. 5a; Supplementary Data 2). We also measured the corresponding protein expression for all but 8 of these phosphopeptides. Only one protein in our data set, fatty acid synthase (FAS), had a significant change in both phosphorylation (at pSer2191) and protein expression, both of which were decreased in the high BCKA condition. This suggests that changes in phosphorylation reflect altered kinase/phosphatase activity rather than changes in protein levels for all of the significantly upregulated and downregulated phosphopeptides other than the one from FAS.

While the majority of modulated phosphosites currently have no functional assignment^29^, gene ontology (GO) enrichment analysis revealed that phosphoproteins regulated by elevated cardiac BCKA exposure are significantly overrepresented in regions such as the actin cytoskeleton, actomyosin, and contractile actin filament bundle (Fig. 5b; Supplementary Data 3). There was also significant enrichment in process terms such as positive regulation of protein polymerization and ventricular cardiac muscle cell action potential. Notably, the cardiac voltage-gated sodium channel Na_v_1.5 (SCN5A), the cardiac ryanodine receptor (RYR2A), and their scaffolding protein, alpha-1 syntrophin (SNTA1) each showed increased phosphorylation (Supplementary Data 2). These data suggest that in addition to promoting protein synthesis, exposure to elevated levels of BCKA may coordinately regulate the actions of proteins involved in myofilament assembly, cardiac conduction, and contractility.

Among the phosphosites with an annotated function, the activating regulatory sites of the mitogen-activated protein kinase (MAPK), MAPK14/p38α MAPK, Thr180, and Tyr182, were significantly downregulated upon exposure of the isolated heart to high levels of BCKA, with no change in protein expression (Fig. 5a; Supplementary Data 2). In contrast, phosphorylation on Ser255, a stimulatory residue of the serine/threonine-protein kinase A-Raf, which activates MEK-ERK signaling^30^, was significantly upregulated (Supplementary Data 2). These data suggest that exposure to elevated BCKA levels may invoke cardiac ERK signaling while dampening signaling through MAPK14/p38α MAPK. Consistent with this interpretation, there was lower phosphorylation of the p38α MAPK target site on STAT1 (Ser727) and significantly less phosphorylation on Thr304 of MAPKAP2, the downstream p38 MAPK Kinase (Supplementary Data 2). We also detected significantly higher phosphorylation of defined MEK-ERK-regulated phosphosites on the proteins Rcsd1 (Ser120), Cttn (Ser369), and Mapt (Ser510). Notably, among these ERK-regulated proteins, Rcsd1 interacts with the F-actin capping protein CapZ to regulate actin filament assembly^31^.

To further define the nature of the BCKA-regulated phosphoproteome, we next conducted a motif scan using the flanking regions (± seven amino acids) of the upregulated and downregulated phosphosites. The downregulated site list was limited to the top 60 sites ranked using P-value. These analyses highlighted considerable homology among the regulated phosphosites. An R-x-x-pS motif was present in 21 of the 60 downregulated phosphosites (Fig. 5c) and a pS/T-P motif was present in 21 of the 35 upregulated phosphosites (Fig. 5d). The R-x-x-pS motif found in downregulated phosphosites is a kinase substrate motif for both calmodulin-dependent protein kinase II (CAMKII) and Protein kinase A (PKA)^32,33^. This sequence has also been identified as a potential 14-3-3 domain-binding motif^34^. The pS/T-P motif present in upregulated phosphosites is a kinase motif for ERK1/2^33^.

We next examined the connectivity of the phosphoprotein networks influenced by cardiac exposure to high levels of BCKA using STRING v11^35^. Analysis of known protein-protein interactions among the downregulated phosphoproteins identified four distinct nodes with four or more known interactors and four nodes with two interactors (Fig. 5e). These nodes represent a range of distinct biological processes, spanning p38α MAPK signaling (MAPK14, MAPKAPK2, STAT1, CTTNA1; red), the 26S proteasome (PSMD4, PSMD11, UBE4B, HSPA4, HSPB6, TP53BP1; blue), lipid metabolism (LIPE, PLIN5; yellow), and purine nucleotide cycling (AMPD2, ADSSL1; green). Analysis of the smaller list of upregulated phosphoproteins yielded two small nodes (Fig. 5f) representing components of the cardiac action potential (SCN5A, RYR2, SNTA1; purple) and actin reorganization/myosin II assembly (CTTN, SVIL; pink).

Finally, given our demonstration of increased phosphorylation of the mTOR target and key protein translation regulator 4E-BP1 in response to high levels of BCKA (Fig. 4b), we performed a manual screen for defined regulatory phosphosites in the mTOR signaling pathway using a significance threshold of 1.5 fold change and *P* < 0.05. The only differential regulation of the mTOR pathway was an increase in phosphorylation of Ser859 of the Regulatory-associated protein of mTOR (Raptor) in response to high levels of BCKA. This phosphosite is directly regulated by ERK, with phosphorylation leading to increased mTOR activity and phosphorylation of 4E-BP1^36^. These data suggest that BCKA-mediated activation of RAF-MEK-ERK signaling in the heart likely underlies the increased phosphorylation of 4E-BP1 to drive protein synthesis.

## Discussion

**Fig. 6:**
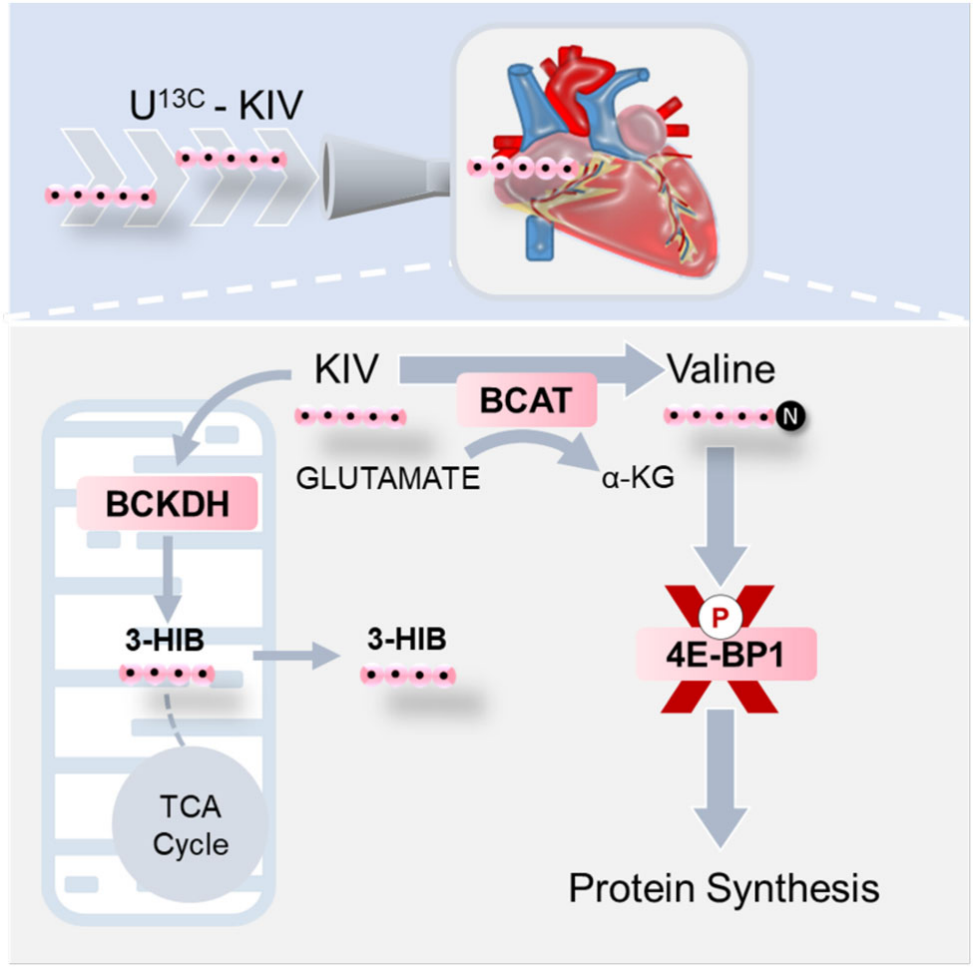
Schematic of KIV fate in the heart. Stable isotope-resolved metabolomics in isolated hearts perfused with [U-^13^C] KIV demonstrates that the major fates of KIV are not oxidation to TCA cycle intermediates but rather reamination to valine and conversion to 3-hydroxyisobutyrate (3-HIB). Acute exposure of isolated hearts to increased supply of BCKA is sufficient to promote inhibitory phosphorylation of the translational repressor 4E-BP1 and increase protein synthesis.

In this study, we demonstrate that the heart preferentially reaminates BCKA to BCAA in a BCAT-dependent manner. In the isolated heart, the degree of reamination is not influenced by increasing BCKDH enzyme activity via treatment with the BDK inhibitor, BT2. Moreover, an increased supply of BCKA to the isolated heart results in increased protein synthesis, likely through phosphorylation and inactivation of the translation repressor protein 4E-BP1, highlighting a potential pathophysiologic role for cardiac BCKA reamination (see Fig. 6). We also present evidence that low levels of expression of SLC25A44 in the heart facilitate the accumulation of BCAA derived from reamination in the cytosol.

Preferential reamination of BCKA rather than oxidation allows BCAA to contribute to cellular processes other than energy metabolism, such as protein synthesis. A number of studies have demonstrated that supplemental BCKA, both in the absence of dietary BCAA or in the setting of a complete diet, can support growth in rats, likely via nitrogen sparing that occurs from reamination of BCKA to BCAA and decreased protein turnover^37^. While this pro-growth effect of BCKA may be beneficial in diseases where increased proteolysis contributes to disease progression, such as chronic renal failure^37^, chronic increases in BCKA delivery to tissues may also promote pathologic hypertropy.

Reamination of BCKA to BCAA may make positive contributions to cardiovascular health under normal circumstances, for example by fueling physiologic cardiac hypertrophy in response to exercise. However, in circumstances of metabolic dysregulation such as obesity, insulin resistance, and diabetes, BCAA and BCKA levels increase chronically due in part to suppression of their catabolism in adipose tissue and liver^15^, and possibly also via a higher rate of the production of these metabolites by the gut microbiome of obese individuals^38^. Early stages of heart failure, which are characterized by compensatory ventricular hypertrophy to adapt to increased hemodynamic stress, are also associated with increases in circulating BCAA and BCKA in humans and animal models^11,39,40^. Under these pathologic conditions, preferential diversion of BCKA to the reamination pathway may contribute to constitutively high rates of protein synthesis leading to cardiac hypertrophy and adverse structural remodeling.

Our data suggest that elevated levels of BCKA rapidly signal to activate protein synthesis in the heart via promoting the phosphorylation and inactivation of the transcriptional repressor protein 4E-BP1. Hypophosphorylated 4E-BP1 suppresses cap-dependent translation by binding to eIF-4E, the limiting component of the translation initiation complex^41^. Phosphorylation of 4E-BP1 promotes the dissociation of the 4E-BP1-eIF-4E complex, leading to the rapid formation of the translation initiation complex and subsequent cap-dependent protein translation.

In addition, our survey of changes in the cardiac phosphoproteome in response to exposure to elevated BCKA provides the first broad evaluation of BCKA-mediated signal transduction in the heart. This unbiased analysis highlighted an array of relevant biological processes that may be impacted in response to obesity-related or heart failure-related elevations in circulating BCKA. Elevated BCKA levels promote MEK-ERK signaling while blunting signal transduction through p38α MAPK. Activation of MEK-ERK signaling in the heart has been shown to promote cardiac hypertrophy^42^. Moreover, increased phosphorylation of Raptor on the ERK-regulated phosphosite Ser859 provides a likely mechanism to account for the increased phosphorylation of 4E-BP1 and activation of protein translation observed in hearts acutely exposed to high levels of BCKA^36^ (Fig. 4). The role of p38α MAPK in hypertrophy is less clear, but there are some studies showing that loss of cardiac p38α MAPK signaling in mice can enhance the development of cardiac hypertrophy^43,44^. Cardiac p38α MAPK also regulates contractility by suppressing the myofilament calcium response^45^. Taken together, these data suggest that the BCKA-mediated shift in MEK-ERK versus p38α MAPK signaling drives protein translation, implying that chronic increases in BCKA could lead to the development of pathologic cardiac hypertrophy and impaired cardiac contractility. Additional studies are warranted to determine the role of p38α MAPK and ERK in mediating the effects of BCKA on cardiac protein synthesis and to directly assess the impact of BCKA on cardiac conduction and contractility. It also remains to be determined whether the effects of BCKA exposure on these signaling pathways are directly mediated by BCKA or require BCKA metabolism to BCAA, 3-HIB, or another metabolite.

Recent studies have investigated the role of altered cardiac BCAA metabolism in cardiovascular disease models. All of these studies suggest that decreased BCAA catabolism in the diseased heart, secondary to downregulation of BCAA oxidative machinery, contributes to the accumulation of BCKA and BCAA that leads to deterioration of cardiac function^11–13^. In support of this hypothesis, whole-body activation of BCKDH via administration of BT2 has been reported to augment cardiac BCAA catabolism and improve cardiac phenotypes^11,12^. While BT2-treated mice demonstrated delayed heart failure progression, they also exhibited significant decreases in circulating BCAA and BCKA. We and others have observed that BCKDH is hyperphosphorylated and in a primarily inactive state in the heart in vivo^6,46^. Moreover, studies in the isolated heart have shown that BCAA oxidation is not a major contributor to myocardial ATP production^20,47^. This observation was recently confirmed in human hearts, where it was determined that BCAA contributes less than 5% of total carbon combustion^48^. Thus, the low rate of BCAA oxidation at baseline makes it unlikely that further decreases in BCAA oxidation will impact cardiac patholog*y*. Given our finding that BCKA is preferentially reaminated to BCAA, it is likely that lowering of circulating BCAA and BCKA levels caused by BT2 treatment, and the resultant reduced delivery of these substrates to the heart plays a more important role in the salutary effects of BT2 treatment than activation of BCAA and BCKA catabolic flux in heart tissue per se.

Our study also found that an important fate of ^13^C-labeled KIV in the heart is its efficient conversion to 3-HIB, but not to succinate or other TCA cycle intermediates. 3-HIB is generated from 3-hydroxyisobutyryl CoA mid-way through the valine catabolic pathway. Complete oxidation of valine involves the further catabolism of 3-HIB to propionyl CoA, methymalonyl CoA, and succinyl CoA, the latter serving as an entrée point to the TCA cycle. Our findings suggest that flux from 3-HIB through one or more of the distal reactions of the valine catabolic pathway is somehow limited in the isolated heart preparation. Interestingly, 3-HIB and its immediate product methylmalonate semialdehyde are the only intermediates of mitochondrial BCAA catabolism that are not CoA-modified after oxidative decarboxylation by BCKDH, potentially allowing these metabolites to be transported out of the mitochondria and the cardiomyocyte. The strong increase in 3-HIB labeling observed with exposure of the isolated heart to labeled KIV may suggest that the partial oxidation of valine and KIV to 3-HIB offers an “escape valve” for excess carbon that might otherwise be used to fuel pathologic rates of total protein synthesis leading to cardiac hypertrophy. It should also be noted that 3-HIB has been ascribed a possible role in the development of insulin resistance, via its activity to promote trans-endothelial lipid transport and tissue lipid accumulation in skeletal muscle^16^. If this mechanism also applies to the heart, chronic increases in BCAA and BCKA as found in obesity may drive production of 3-HIB, in turn leading to cardiac lipid deposition, contributing to the development of cardiovascular disease. Consistent with such a mechanism, we have recently found that dietary BCAA restriction reduces elevated cardiac triglyceride stores in Zucker obese rats^8^.

One possible explanation for the preferential reamination of BCKA to BCAA in the heart is the low activity of the BCKDH complex relative to other tissues^6,46^. In addition, we hypothesized that low expression of the recently described mitochondrial BCAA transporter, SLC25A44, in the heart, could regulate BCKA reamination by preventing entry of BCAA into the mitochondria for oxidation. Consistent with this hypothesis, we found that *Slc25a44* expression negatively correlated with reamination of KIV across rat tissues, with the lowest expression found in the heart, and that overexpression of SLC25A44 in the heart reduced KIV reamination. However, the reduced reamination was not due to increased mitochondrial KIV oxidation as we expected, but rather to increased export of valine out of cardiomyocytes and into the perfusion media. This likely occurred due to a coordinate increase in expression of the BCAA plasma membrane transporter SLC7A5 (Lat1). Supporting this idea, we found a strong correlation between *Slc25a44* and *Slc7a5* mRNA expression in rat hearts, but not in other tissues such as the liver. Although SLC7A5 is thought to mediate cellular BCAA import, in vitro studies reveal that intracellular loading with leucine promotes leucine efflux through this transporter^49,50^. Thus, increased uptake of BCKA by the heart in the setting of elevations in their levels, and their subsequent reamination to BCAA may increase cytoplasmic pools of BCAA to be utilized concurrently for cellular export and protein synthesis. The exact mechanism by which SLC25A44 and SLC7A5 are coordinately regulated in the heart remains an area for future investigation.

While our work has identified the fates of BCKA in the heart and provides insight into the effect of cardiac BCKA exposure on the phosphoproteomic landscape, we did not evaluate the effect of these perturbations on cardiac structure or function. Future studies in chronic models of BCKA perturbation are required to determine whether the increase in protein synthesis rates occurring with acute exposure to pathophysiologic levels of BCKA leads to cardiac hypertrophy and impaired function. Likewise, additional efforts should be devoted to determining whether the effects of BCKA to alter the phosphorylation of ion channels translate into changes in cardiac conduction and contractility.

In conclusion, using stable isotope tracing in isolated perfused hearts and in living rats, we show that the heart preferentially reaminates BCKA to BCAA, while also causing widespread changes in the cardiac phosphoproteome, leading to an increase in cardiac protein synthesis. The degree of BCKA reamination across tissues is inversely correlated with expression of the mitochondrial BCAA transporter, SLC25A44, suggesting that tissue-specific BCKA fate may be determined by mitochondrial access or the balance between cellular BCAA import and export. These studies provide a framework for understanding why plasma BCAA and BCKA associate with cardiometabolic diseases.

## Methods

### Animals

Male Wistar rats (250–274 g) were used for the isolated heart perfusion and in vivo studies and were purchased from Envigo. Male C57BL/6J mice from Jackson laboratories were used for over-expression of cardiac SLC25A44 and isolated heart perfusion. Animals were dual housed in a 12 h light:dark cycle and given ad-libitum access to a standard chow diet (TD.7001, Harlan Teklad for rats and PMI 5053, Picolab for mice) and water. SLC25A44 and GFP expression plasmids with a CMV promoter containing ITRs were used to prepare recombinant AAV9 by the UMass Gene Therapy Core. Adult 8-week-old male mice were injected intravenously with 100 µl of 5.1 × 10^11^ vector genomes per mouse. All animal procedures were approved by the Duke University Institutional Animal Care and Use Committee and performed according to the ethical guidelines and regulations outlined by the Animal Welfare Act.

### Isolated heart perfusions

Fed male Wistar rats or C57BL/6J mice were anesthetized with 5% isoflurane, and isolated hearts were perfused in the Langendorff mode at 37 °C with non-recirculating perfusate. The hearts were allowed to beat spontaneously throughout the perfusion. All heart perfusions underwent an initial 15-min equilibration period with Krebs Ringer bicarbonate buffer containing 119 mM NaCl, 4.8 mM KCl, 2.6 mM CaCl_2_, 1.2 mM KH_2_PO_4_, 1.2 mM MgSO_4_, 25 mM NaHCO_3_, 11 mM glucose, and 0.05 mM L-carnitine at a flow rate of 12 ml/min for rats or 1.5 ml/min for mice^51–53^. At the end of each perfusion, hearts were immediately freeze-clamped in liquid nitrogen using the Wollenberger technique and stored at −80 °C for further analysis.

#### Heart perfusions with LY3351337

After the 15-min equilibration perfusion, male Wistar rat hearts were perfused for 30 min with Krebs Ringer bicarbonate buffer with the following additions: 3% BSA (fatty acid free; Fisher Scientific), 100 µU/mL insulin, 0.4 mM palmitate (Sigma) bound to BSA, physiologic concentrations of amino acids (Alanine 416 µM, Arginine 137 µM, Asparagine 33 µM, Aspartic Acid 10 µM, Cysteine 15 µM, Glutamic Acid 89 µM, Glutamine 483 µM, Glycine 220 µM, Histidine 50 µM, Isoleucine 88 µM, Leucine 134 µM, Lysine 287 µM, Methionine 40 µM, Phenylalanine 60 µM, Proline 112 µM, Serine 142 µM, Threonine 164 µM, Tryptophan 55 µM, Tyrosine 70 µM, Valine 179 µM), 100 μM [U-^13^C]KIV (Cambridge Isotopes), and either DMSO (Veh) or 40 μM LY3351337 (Eli Lilly Co.).

#### Heart perfusions with BT2

Male Wistar rats were pre-treated with BT2 (20 mg/kg/d via i.p. injection; Chem-Impex) for three days. Hearts were isolated and, after the 15-min equilibration perfusion, were perfused for 30 min with Krebs Ringer bicarbonate buffer with the following additions: 3% BSA (fatty acid free; Fisher Scientific), 100 µU/mL insulin, 0.4 mM palmitate (Sigma) bound to BSA, physiologic concentrations of amino acids (Alanine 416 µM, Arginine 137 µM, Asparagine 33 µM, Aspartic Acid 10 µM, Cysteine 15 µM, Glutamic Acid 89 µM, Glutamine 483 µM, Glycine 220 µM, Histidine 50 µM, Isoleucine 88 µM, Leucine 134 µM, Lysine 287 µM, Methionine 40 µM, Phenylalanine 60 µM, Proline 112 µM, Serine 142 µM, Threonine 164 µM, Tryptophan 55 µM, Tyrosine 70 µM, Valine 179 µM), 100 μM [U-^13^C]KIV (Cambridge Isotopes), and either DMSO (Veh) or 400 μM BT2 (Chem-Impex).

#### Heart perfusions with puromycin

After the 15-min equilibration perfusion, male Wistar rat hearts were perfused for 30 min with Krebs Ringer bicarbonate buffer with the following additions: 3% BSA (fatty acid free; Fisher Scientific), 100 µU/mL insulin, 0.4 mM palmitate (Sigma) bound to BSA, physiologic concentrations of amino acids (Alanine 416 µM, Arginine 137 µM, Asparagine 33 µM, Aspartic Acid 10 µM, Cysteine 15 µM, Glutamic Acid 89 µM, Glutamine 483 µM, Glycine 220 µM, Histidine 50 µM, Isoleucine 88 µM, Leucine 134 µM, Lysine 287 µM, Methionine 40 µM, Phenylalanine 60 µM, Proline 112 µM, Serine 142 µM, Threonine 164 µM, Tryptophan 55 µM, Tyrosine 70 µM, Valine 179 µM), 1 μM puromycin (Sigma) and either no branched-chain α-ketoacids (BCKA), concentrations of BCKA found in the plasma of lean rats [α-ketoisovalerate (KIV) 12 μM; α-ketoisocaproate (KIC) 15 μM; α-keto-β-methylvalerate (KMV) 7 μM; all purchased from Sigma] or concentrations of BCKA found in the plasma of obese rats (KIV 24 μM; KIC 30 μM; KMV 14 μM)^5^.

#### Mouse heart perfusions

Four weeks after injection, hearts were isolated from male C57BL/6J treated with either AAV9-CMV-GFP or AAV9-CMV-SLC25A44 and, after the 15-min equilibration period, were perfused for 30 min with Krebs Ringer bicarbonate buffer with the following additions: 3% BSA (fatty acid free; Fisher Scientific), 100 µU/mL insulin, 0.4 mM palmitate (Sigma) bound to BSA, physiologic concentrations of amino acids (Alanine 416 µM, Arginine 137 µM, Asparagine 33 µM, Aspartic Acid 10 µM, Cysteine 15 µM, Glutamic Acid 89 µM, Glutamine 483 µM, Glycine 220 µM, Histidine 50 µM, Isoleucine 88 µM, Leucine 134 µM, Lysine 287 µM, Methionine 40 µM, Phenylalanine 60 µM, Proline 112 µM, Serine 142 µM, Threonine 164 µM, Tryptophan 55 µM, Tyrosine 70 µM, Valine 179 µM), and 100 μM [U-^13^C]KIV (Cambridge Isotopes).

### Validation of LY3351337 as a BCAT inhibitor

The ability of LY3351337 to inhibit BCAT was tested using a 10-point dose-response curve (20–0.001 µM) in HEK293 and L6 cells. Cells were treated with LY3351337 for 15 min prior to a 2-h incubation in media containing U-^13^C,^15^N-L-leucine. BCAT activity was determined by quantifying ^15^N glutamate in cell lysates by LC-MS on a rapidFire system coupled to a Sciex 6500 triple quadrupole MS operated in positive heated electrospray mode with SRM detection. The internal standard was DL-glutamic 2,4,4-d3 acid (CDN Isotopes). Inhibitory activity of LY3351337 for BCAT1 and BCAT2 was determined in vitro using purified rat enzymes. Rat BCAT1 and BCAT2 cDNA was purchased from GE Healthcare and the nucleotide sequences were inserted into a pET21d vector (Novagen) with an N-terminal HIS tag. Enzymes were expressed in BL21 (DE3) bacteria (Novagen) then purified from cell pellets using HisPur Ni-NTA resin (Thermo Scientific). BCAT activity was determined by measuring glutamate generation by LC-MS in reaction mixtures containing 2 nM of purified BCAT1 or BCAT2, L-leucine (900 µM), and αKG (400 µM) in a 50 mM phosphate buffer (pH 7.4) containing 0.5 mM octylglucoside, 10 mM DTT, 0.005%BSA, and 0.5 µM PLP. To verify the specificity of LY3351337, purified aspartate transaminase (AST) and alanine transaminase (ALT) were incubated with LY3351337, and activity determined in a clinical analyzer, the transaminase inhibitor aminooxyacetic acid (AOA) was used as a positive control.

### In vivo stable isotope tracing studies

#### Kinetic studies

Thirty minutes after a single injection of BCAT inhibitor, LY3351337 (10 mg/kg i.p., Eli Lilly Co.), or vehicle (DMSO, Sigma), male Wistar rats received an i.p. injection of [U-^13^C]KIV (100 mg/kg, Cambridge Isotopes), followed by blood sampling from the tail vein at 2, 5, 10, 15, 30, and 60 min.

#### Tissue ^13^C enrichment studies

A separate cohort of male Wistar rats was treated with LY3351337 (10 mg/kg i.p., Eli Lilly Co.) or vehicle (DMSO, Sigma) and received an i.p. injection of [U-^13^C]KIV (100 mg/kg, Cambridge Isotopes) 30 min later. Tissues were harvested 30 min after the [U-^13^C]KIV injection and immediately freeze-clamped in liquid nitrogen and stored at −80 °C until further analysis.

### Metabolite profiling

The targeted amino acid, organic acid, and branched-chain α-ketoacid analyses were performed with internal standards as previously described in frozen heart samples^2,6,54^. For 3-HIB measurements, 100 µl of tissue homogenate was prepared at 50 mg of wet tissue per 1 ml of homogenate in 50% acetonitrile/0.3% formic acid in water were spiked with an isotopically labeled internal standard d6-2-hydroxyisobutyric acid (2-HIB) (CDN Isotopes); 100 µl of water were added to the samples, followed by centrifugation. The supernatants were extracted with acidified ethyl acetate, dried under nitrogen, reconstituted in water, and analyzed by LC-MS/MS^55^.

### Stable isotope-resolved metabolite profiling

Frozen tissues were pulverized under liquid nitrogen using a mortar and pestle. Metabolites were then extracted using sequential 500 μl additions of −20 °C MeOH, chilled water, and chloroform. After each addition, tissue lysates were prepared with a Tissue Lyser (Qiagen) for 60 s at 30 Hz. Similarly, plasma metabolites (20 μL) were extracted by sequential 500 μL additions of −20 °C MeOH, chilled water, and chloroform. After each addition, samples were vortexed for 30 s. Tissue and plasma extracts were then centrifuged at 4 °C and 14,400 × *g* for 20 min and the clarified aqueous phase was transferred to a fresh Eppendorf and stored in −80 °C until processing for GC-MS analysis.

For GC-MS analysis, the extracted tissue and plasma metabolites were dried under N_2_ gas-flow at 37 °C using an evaporator. Amino and organic acids were derivatized via methoximation and silylation as previously described^56^. Briefly, metabolites were resuspended in 25 μl of methoxylamine hydrochloride (2% (w/v) in pyridine) and incubated at 40 °C for 90 min on a heating block. After brief centrifugation, 35 μl of MTBSTFA + 1% TBDMS was added and the samples were incubated at 60 °C for 30 min. GC-MS analysis was performed on an Agilent 7890B GC system equipped with an HP-5MS capillary column connected to an Agilent 5977 A Mass Spectrometer^57^. Mass isotopomer distributions were obtained by integration of ion chromatograms^58^ and corrected for natural isotope abundances^59^. Metabolites monitored were valine (*m/z* 288–295); leucine (*m/z* 274–280); KIV (*m/z* 202–209); 3-HIB (*m/z*: 275–280); citrate (*m/z* 459–467); and succinate (*m/z* 289–295).

### KIV reamination and 3-HIB formation rate determination

The concentration of [U-^13^C]KIV-derived valine, denoted valine_M+5_, in the effluent perfusate was determined by stable isotope dilution with ^15^N-valine as internal standard (Sigma Aldrich; Cat. 490172). Briefly, 10 μl of 100 μM ^15^N-valine (M + 1) was spiked into 100 μl of perfusate. Proteins were precipitated with 1 ml acetonitrile (LC-MS grade), centrifuged at 15,000 × *g* for 5 min at 4 °C, and the supernatants were dried under N_2_ gas-flow at 60 °C. Derivatization, GC-MS analysis, and correction of raw data for natural abundances were performed as previously described^56–59^. The concentration of valine_M+5_ (in μM) was equal to: $\frac{{{\mathrm{corrected}}\,{\mathrm{valine}}_{M + 5}}}{{{\mathrm{corrected}}\,{\mathrm{valine}}_{M + 1}}} \times 10$. Standard curves were also performed to ensure linearity in the range of perfusate valine_M+5_ concentrations. The concentration of 3-HIB_M+4_ (in μM) was equal to: $\frac{{{\mathrm{corrected}}\,3 - {\mathrm{HIB}}_{M + 4}}}{{{\mathrm{corrected}}\,3 - {\mathrm{HIB}}_{M + 0}}} \times \left[ {3{\mathrm{HIB}}_{M + 0}} \right]\left( {{\mathrm{\mu}} {\mathrm{M}}} \right)$, where the unlabeled concentration of 3-HIB (in μM) was determined by LC-MS as described above. The reamination rate of KIV (μmol/min) was determined as: $[{\mathrm{valine}}_{M + 5,\,{\mathrm{perf}}}] \left(\frac{\mu{\mathrm{mol}}}{L}\right)\times {\mathrm{{perfusate}}}\; {\mathrm{rate}} \left(\frac{L}{\min}\right)+[{\mathrm{valine}}_{M + 5,\,{\mathrm{tissue}}}] \left(\frac{\mu{\mathrm{mol}}}{g}\right)\times \frac{{\mathrm{heart}}\;{\mathrm{weight}}(g) }{{\mathrm{perfusion}}\; {\mathrm{time}}(\min)}$. The formation of 3-HIB (μmol/min) was calculated using the equation above with 3-HIB_M+4_ in place of valine_M+5_. The rate calculation assumes that the efflux rate and enrichments of valine_M+5_ or 3-HIB_M+4_ have achieved a steady-state. Furthermore, it is assumed that the effluxed and tissue valine_M+5_ content is much greater than valine_M+5_ incorporated into protein.

### Cardiac BCKDH activity

Cardiac BCKDH activity was determined as previously described^6^. Briefly, frozen tissue samples were pulverized in liquid nitrogen, then homogenized using a QIAGEN TissueLyser II in 250 μl of ice-cold buffer I (30 mM KPi pH 7.5, 3 mM EDTA, 5 mM DTT, 1 mM α-ketoisovalerate, 3% FBS, 5% Triton X-100, 1 μM Leupeptin). Samples were then centrifuged for 10 min at 10,000 × *g* and 50 μl of supernatant was added to 300 μL of buffer II (50 mM HEPES pH 7.5, 30 mM KPi pH 7.5, 0.4 mM CoA, 3 mM NAD+, 5% FBS, 2 mM Thiamine Pyrophosphate, 2 mM MgCl_2_, 7.8 μM α-keto [1-^14^C] isovalerate) in a polystyrene test tube containing a raised 1 M NaOH CO_2_ trap. Tubes were capped and placed in a shaking water bath at 37 °C for 30 min. The reaction mixture was acidified by injection of 70% perchloric acid followed by shaking on an orbital shaker for 1 h. The ^14^CO_2_ contained in the trap was counted in a liquid scintillation counter.

### Immunoblotting

Tissue lysates used for immunoblotting were prepared in Cell Lysis Buffer (Cell Signaling Technologies) containing protease inhibitor tablets (Roche), phosphatase inhibitor cocktails 2 and 3 (Sigma), and 10 mM PMSF. 50 μg of protein was loaded onto a 4–12% Bis-Tris gel (Novex), subjected to SDS-PAGE, and then transferred onto PVDF membranes. Membranes were blocked and then probed with the appropriate antibodies. All primary antibodies were used at a concentration of 1:1000. Secondary antibodies were diluted 1:10000. The following antibodies were used: S6 (Cell Signaling Technologies #2317), phospho-S6 (Ser 235/236) (Cell Signaling Technologies #4858), 4E-BP1 (Cell Signaling Technologies #9644), phospho-4E-BP1 (Thr 37/46) (Cell Signaling Technologies #2855), pan-actin (Cell Signaling Technologies #8456), puromycin (EMD Millipore #MABE343), β-tubulin (Sigma Aldrich, T8328), Alexa-Fluor Plus 800 (Thermo Fisher, A32735), and Alexa-Fluor Plus 594 (Thermo Fisher, A32742). Immunoblots were developed using a Li-Cor Odyssey CLx and quantified using the Li-Cor software.

### Quantitative (phospho)proteomics

#### Sample preparation

Heart tissue was lysed by probe sonication in 500 μl of 5% SDS (w/v) in 50 mM triethylammonium bicarbonate buffer, pH 8.5 (TEAB) by probe sonication followed by BCA assay. Four hundred and fifty micrograms of each sample was normalized to 100 μl of lysis buffer followed by spike-in of bovine casein, the addition of 10 mM DTT, and reduction at 80 °C for 10 min. After cooling, samples were alkylated with 25 mM iodoacetamide in the dark for 30 min at room temperature. SDS was removed, and samples were digested with 20 μg of Sequencing Grade Modified Trypsin (Promega) using an S-trap mini device (Protifi) according to instructions (digestions at 47 °C for 2 h). Recovered peptides were lyophilized to dryness. Lyophilized peptides were reconstituted in 100 μl of 200 mM TEAB buffer and peptides were labeled with 41 μl of TMT10 reagents (lot# UL292365). After 2 h, reactions were quenched with 5% hydroxylamine for 15 min and all samples were mixed and lyophilized.

TMT-labeled peptides were fractionated by high pH reversed-phase (HPRP) chromatography. Briefly, peptides were reconstituted in 400 μl of 20 mM ammonium formate, and 125 μl was fractionated using a 2.1 mm × 5 cm BEH C18 column (Waters) and Waters ACQUITY iClass UPLC. Separations utilized a flow rate of 0.4 ml/min and column temperature of 55 °C, and mobile phases consisted of 20 mM ammonium formate, pH 10 (MPA), and neat MeCN (MPB). Separations used a gradient as follows: 0 min, 3% B; 3 min, 7% B; 50 min, 50% B, 51 min, 90% B; 55 min, 90% B; 56 min, 3% B; 60 min, 3% B. Forty-eight equal fractions were collected over 52 min into a 96-well plate containing 10 μl of 20% TFA per well. This analysis was repeated 2 times total for fractionation of 2 mg peptides. The first 3 fractions of each injection were excluded, and the remaining samples were re-concatenated into 12 fractions. Approximately 5 μg of each fraction was separately aliquoted for analysis of the unenriched proteome, and samples were lyophilized.

For phosphopeptide enrichment, ~200 μg of each of the 12 HPRP fractions were reconstituted in 80% MeCN/1% TFA containing 1 M glycolic acid (buffer A), phosphopeptides were enriched using GL Sciences p10 TiO tips as previously described^60,61^. After loading, tips were washed twice with buffer A followed by 2 times with 80% MeCN/1% TFA before elution with 20% MeCN/5% aqueous ammonia. After the addition of neat formic acid, samples were lyophilized. Finally, peptides were desalted using C18 State Tips, lyophilized, and reconstituted in 12 μl of 10 mM citrate in 1% TFA/2% MeCN. The unenriched fractions were reconstituted in 10 μl of 1% TFA/2% MeCN.

#### Quantitative mass spectrometry

One-dimensional liquid chromatography, tandem mass spectrometry (1D-LC-MS/MS) was performed on 4.5 μl of each of the phospho-enriched fractions or on 0.75 μg of unenriched fractions. Samples were analyzed using a nanoACQUITY UPLC system (Waters) coupled to an Exploris 480 high-resolution accurate mass tandem mass spectrometer (Thermo) via a nanoelectrospray ionization source and FAIMS Pro interface. Samples were first trapped on a Symmetry C18 180 μm × 20 mm trapping column (5 μl/min at 99.9/0.1 v/v H2O/MeCN) followed by an analytical separation using a 1.7 μm Acquity HSS T3 C18 75 μm × 250 mm column (Waters) with a 90 min gradient of 5 to 30% MeCN with 0.1% formic acid at a flow rate of 400 nl/min and column temperature of 55 °C. Data collection was performed in data-dependent acquisition (DDA) mode with 3 FAIMS compensation voltages (−40, −60, and −80). Each CV used a 60,000 resolution full MS scan from *m*/*z* 375 to 1600 with a normalized AGC target of 300%, peptide monoisotopic peak determination, an intensity threshold of 5E3 ions, precursor fit of 70% with 0.7 m/z fit windows, selection of charge state of 2–5 and 40 s dynamic exclusion. MS/MS used a top6 method for each CV with 30,000 resolution and TurboTMT enabled, an isolation width of 0.7 *m*/*z*, NCE of 36, AGC target of 300%, and maximum IT of 120 ms. Advanced precursor determination was enabled. The analysis of unenriched samples used a similar method except that a 1 s total scan time was used for each CV and MS/MS used an auto IT. Raw data have been deposited in the MassIVE database https://massive.ucsd.edu and is publicly available under the dataset identifier MSV000086199.

#### Data analysis

Data were analyzed using Thermo Proteome Discoverer 2.4. Database searching was performed against the Uniprot database (reviewed and unreviewed) with rattus taxonomy (downloaded on 05/28/20; 29,950 entries) including bovine casein isoform protein sequences. Searches used the MSFragger PD2.4 node and MSFragger 2.4^62^ with default closed search parameters, trypsin specificity with up to 2 missed cleavages, no recalibration, fixed carbamidomethylation (Cys) and TMT10 (Lys and peptide N-terminal), and variable phosphosphorylation (Ser, Thr, and Tyr). Percolator and imp-ptmRS nodes were used for FDR determination and site localization. The processing node of the reporter ions quantifier used a 10 ppm integration tolerance, and the consensus node used correction for isotopic impurity (for TMT lot# UKL292365), a co-isolation threshold of 50%, and an S/N threshold of 5. All high confidence peptides (including shared peptides) were considered for quantification of phosphopeptides, whereas unique + razor was used for the unenriched fractions. Low abundance resampling was used for the imputation of missing values. Normalization to total peptide intensity and ANOVA were used for statistical testing in PD. Following initial QC, a single outlier (low BCKA sample) was removed and the data re-analyzed.

### Reverse transcription real-time quantitative-PCR analysis

RNA was extracted from tissues using an RNeasy kit from QIAGEN. RNA was reverse transcribed using the Bio-Rad iScript cDNA synthesis kit or High-Capacity cDNA Reverse Transcription Kit (Applied Biosystems). qPCR was performed with Applied Biosystems TaqMan® gene expression assays for *Bcat1* (Rn00568471_m1), *Bcat2* (Rn00574455_m1), *Slc25a44* (Rn01769831_m1) and *rPLP0* (Rn03302271_gH) and PowerUp SYBr Green Master Mix (Applied Biosystem) for all BCAA transporters, 60S acidic ribosomal protein P0 (*Rplp0*), and Peptidylprolyl Isomerase A (*Ppia*) on a QuantStudio 6 Flex Real-Time PCR system (Applied Biosystems). Each sample was run in duplicate and AAV-treated mouse samples were normalized to *Ppia*, while all other samples were normalized to *Rplp0*. Primers used for the determination of BCAA transporters in mouse hearts following AAV-treatment or rat tissue correlations of *Slc7a5* to *Slc25a44* can be found in Supplementary Table 1.

### Statistical analyses

All data are expressed as mean ± SEM and were analyzed using a paired Student’s *t*-test or one-way ANOVA with Tukey’s HSD post-hoc test where appropriate. Spearman correlation was used to correlate tissue ^13^C concentrations and gene expression. A *p-value* less than 0.05 was considered statistically significant.

### Reporting summary

Further information on research design is available in the Nature Research Reporting Summary linked to this article.

## Supplementary information

Supplementary Information

Description of Additional Supplementary Files

Supplementary Data 1

Supplementary Data 2

Supplementary Data 3

Reporting Summary

## Data Availability

Raw (phospho)proteomics datasets generated during and/or analyzed during this study are available in the MassIVE database, https://massive.ucsd.edu under the dataset identifier MSV000086199. Metabolomics data is available at the NIH Common Fund’s National Metabolomics Data Repository (NMDR) website, the Metabolomics Workbench, https://www.metabolomicsworkbench.org where it has been assigned Project ID (PR001070). The Metabolomics Workbench is supported by NIH grant U2C-DK119886. Source data are provided with this paper. Any additional data supporting the findings from this study are available from the corresponding authors upon reasonable request. Source data are provided with this paper.

## Source data

Source Data
