# Supplementary material for: Branched-chain α-ketoacids are preferentially reaminated and activate protein synthesis in the heart
Source: Nat Commun. 2021 Mar 15;12:1680. doi: 10.1038/s41467-021-21962-2 (PMC7960706; doi:10.1038/s41467-021-21962-2)
Supplement: Supplementary file 1 — Supplementary Information [file 41467_2021_21962_MOESM1_ESM.pdf]

Supplementary Information for

Branched-chain α-ketoacids are preferentially reaminated and activate protein synthesis in the heart

Jacquelyn M. Walejko[1#], Bridgette A. Christopher[1,2#], Scott B. Crown[1#], Guo-Fang Zhang[1,3,4], Adrian Pickar-Oliver[5,6], Takeshi Yoneshiro[7], Matthew W. Foster[8], Stephani Page[1], Stephan van Vliet[1], Olga Ilkayeva[1,9], Michael J. Muehlbauer[1], Matthew W. Carson[10], Joseph T. Brozinick[10], Craig D. Hammond[10], Ruth E. Gimeno[10], M. Arthur Moseley[8], Shingo Kajimura[7], Charles A. Gersbach[5,6,11], Christopher B. Newgard[1,3,4,9], Phillip J. White[1,3,4,9*], Robert W. McGarrah[1,2,4*]

[*]Co-corresponding authors. Emails: phillip.white@duke.edu, robert.mcgarrah@duke.edu

The PDF file includes:

Supplementary Table 1

Supplementary Figures
- Supplementary Figure 1
- Supplementary Figure 2
- Supplementary Figure 3
- Supplementary Figure 4

Supplementary Table 1. Primers used for RT-qPCR		
**Target gene**	**Forward Primers**	**Reverse Primers**
**Mouse**		
Slc7a5	GACATCTGAAGCTGTGGCTG	GCAAGACAAGCCCACAAAGA
Slc7a8	GCCACCCGGGTTCAAGATA	GCCGATGTCAGGTTCTTGG
Slc7a7	TGCAATGGGTGACATTGCTC	GGCATGGAAATGCCAATGGA
Slc7a6	CATAAGAGTGACGCTGTGGC	CAACAGCAATGGGGATGGTC
Slc3a2	ACTGGGGAGCGTACTGAATC	CGGAGAAGATGGTCCGGTAT
Slc3a1	CTCCAGCTGGCACTTTGATG	ACCCTTCGAGAGCCAGAAC
Slc43a1	GCTTCTATTCCAGCCTGTGC	AGGAAGGAGCCAATGGTGAA
Slc43a2	ATGGGGGCTATGAACAGCAT	CCAGTCCATGATGTAGCCGA
Slc7a10	ACTATGTCACGGAGGAGCTG	GGCGACATTGGTGAATGTGT
Slc25a44	TCGCTGCTAACGTACATCCC	AGACAATGTGAGGGCACTCC
Ppia	GCATACGGGTCCTGGCATCTTGTCC	ATGGTGATCTTCTTGCTGGTCTTGC
**Rat**		
Slc7a5	CTCGGCTTCATCCAGATGGG	CATCCTCCGTAGGCGAAGAG
Slc25a44	GGTGGCCCTTTTACCACTTC	TCCATGGGATTGGTGAGGAC
Rplp0	AGCAGGTGTTTGACAATGGC	AACAGTCGGGTAGCCAATCTG

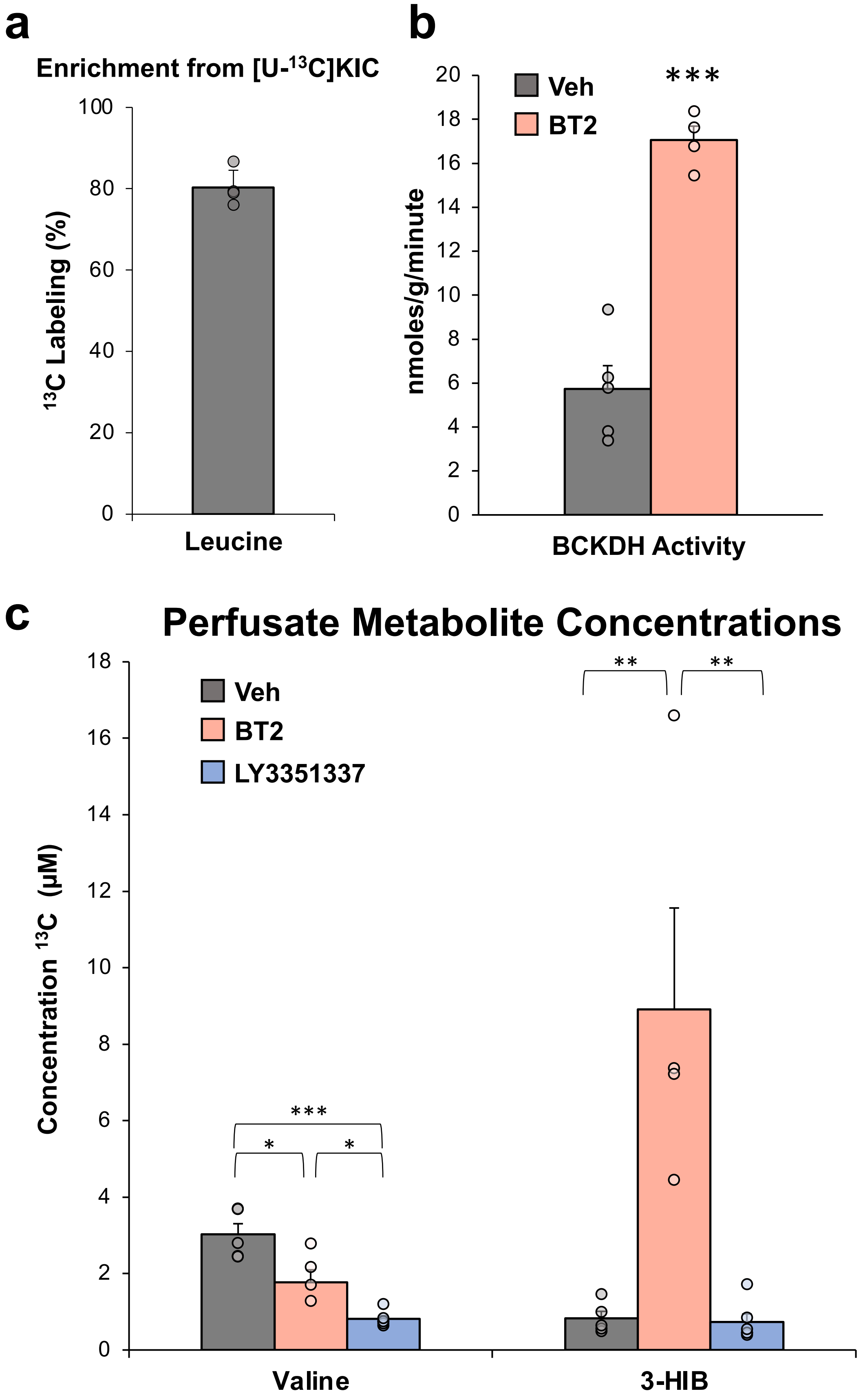

Supplementary Figure 1. Preferential reamination of BCKA to BCAA in the isolated perfused heart. (a) Fractional percent labeling of leucine with ^{13}C in isolated Wistar rat hearts perfused with [1,2-^{13}C]KIC (100μM). N=4. (b) BCKDH activity in perfused hearts from Wistar rats pre-treated for 3 days with BT2 (20 mg/kg/d via i.p. injection; n=4; red) or vehicle (n=4; grey). (c) Absolute amounts of ^{13}C-labeled valine and 3-HIB in perfusate from isolated Wistar rat hearts perfused with [U-^{13}C]KIV in the absence (Veh; n=5; grey) or presence of BDK inhibitor, BT2 (n=4; red) or BCAT inhibitor, LY3351337 (n=6; blue). Data represent mean ± SEM. Statistical differences indicated by two-way, paired Student's t-test (b) or Tukey's HSD pos-hoc test following one-way ANOVA (c): * $P<0.05$, ** $P<0.005$, *** $P<0.0005$.

a

Cell activity		Enzymatic Assay (Rat protein)	
hHEK 293 IC_{50}, nM	rL6 IC_{50}, nM	rBCAT2 IC_{50}, nM	rBCAT1 IC_{50}, nM
138 (n=7)	70.3 (n=4)	1.7 (n=4)	2.1 (n=4)

b

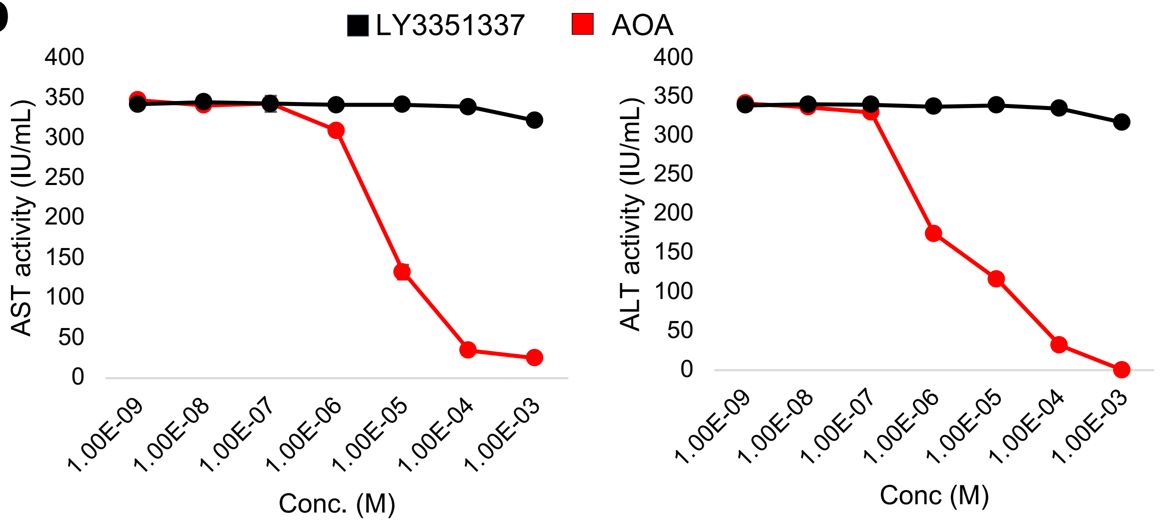

Supplementary Figure 2. LY3351337 is a novel pan –BCAT inhibitor. (a) LY3351337 IC_{50} values in HEK293 (n=7) and L6 (n=4) cells. Inhibitory effect of LY3351337 shown by LY3351337 IC_{50} values for BCAT1 and BCAT2 using purified rat enzymes *in vitro* (right). (b) Aspartate transaminase (AST) and alanine transaminase (ALT) activity following incubation with LY3351337 (black) or the transaminase inhibitor aminooxyacetic acid (AOA) as a positive control (red).

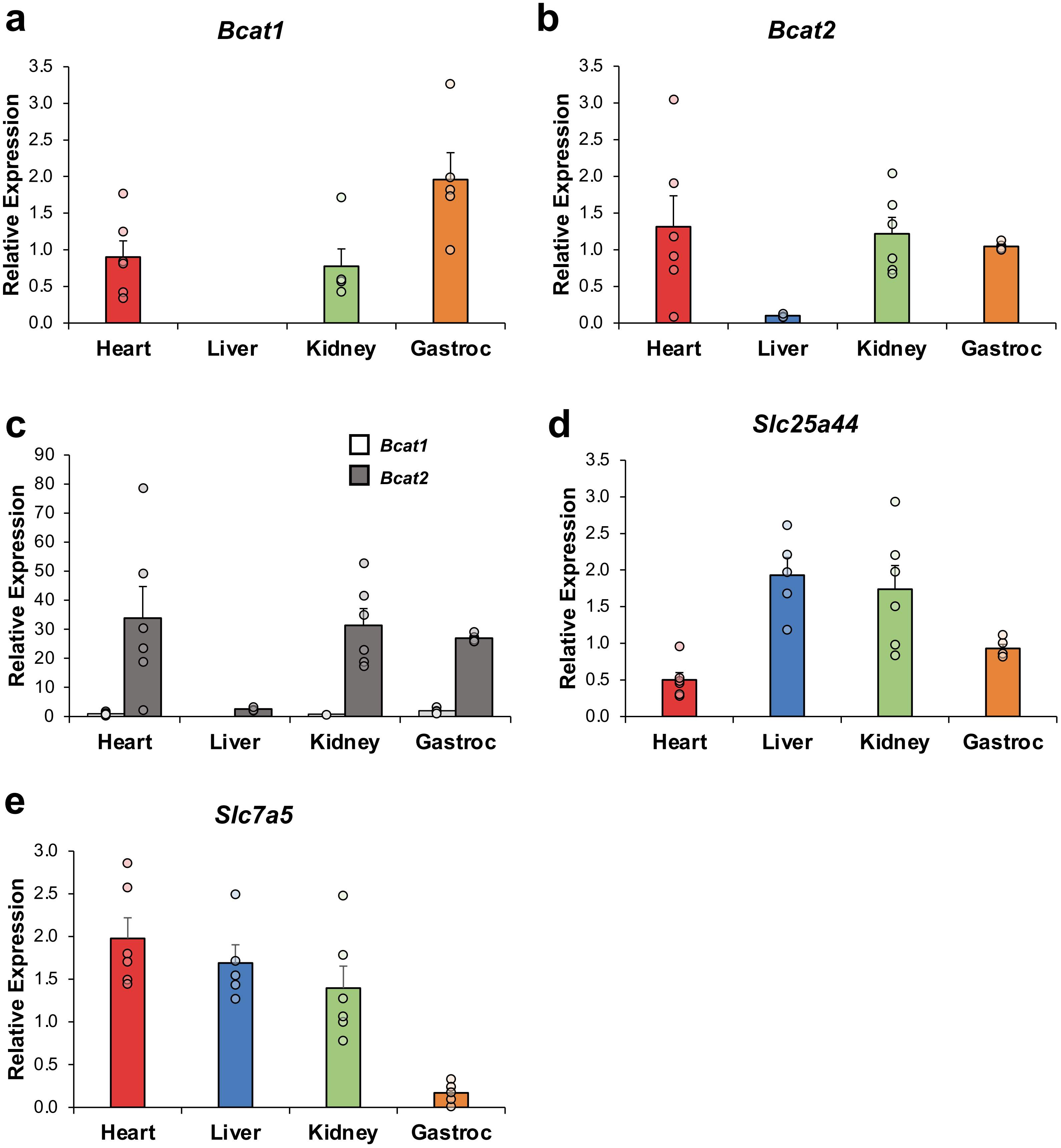

Supplemental Figure 3. Expression of BCAT and BCAA transporters across rat tissue. Heart (n=6; red), liver (n=5; blue), kidney (n=6; green), and gastrocnemius (n=5; orange) mRNA relative expression of *Bcat1* (a), *Bcat2* (b), comparison of *Bcat1* and *Bcat2* across tissues (c), *Slc25a44* (d), or *Slc7a5* (e). Gene expression was normalized to *Rplp0*. Data represent mean ± SEM.

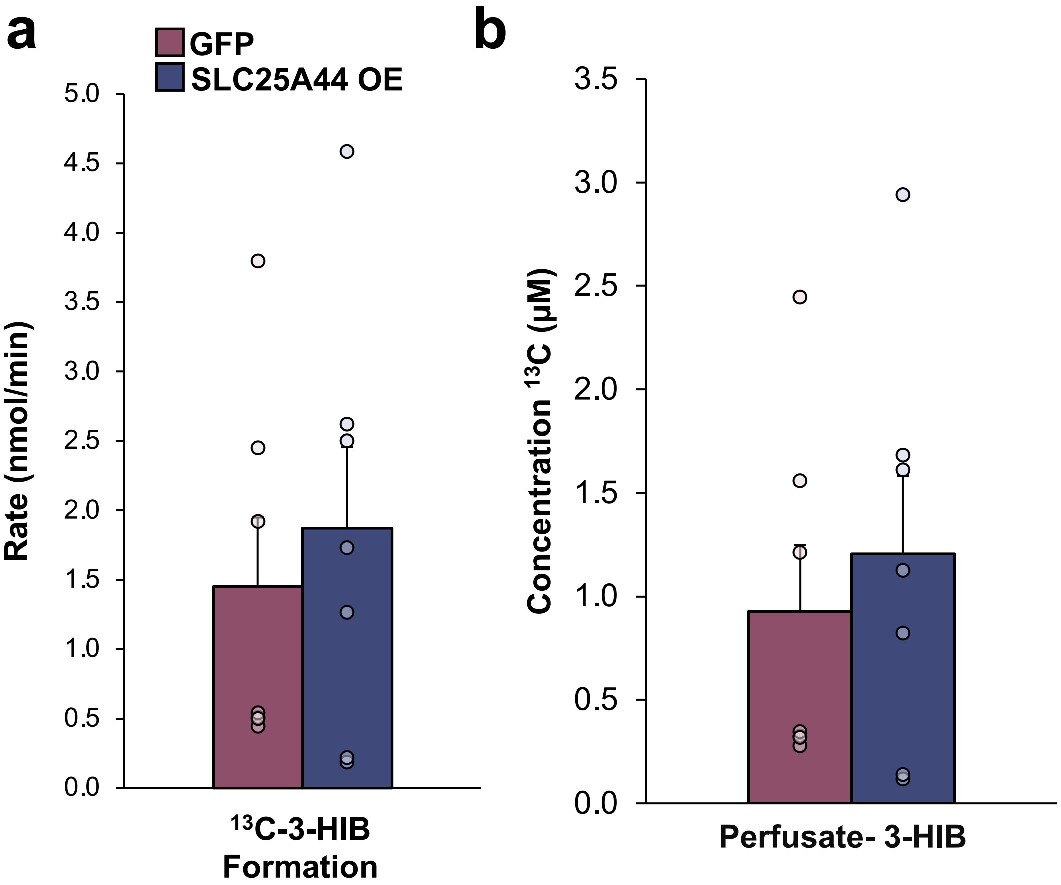

Supplementary Figure 4. Formation of 3-HIB is not altered in mouse hearts following SLC25A44 over-expression. Rate of formation of ^{13}C-labeled 3-HIB from [U-^{13}C]KIV (a) and perfusate metabolite concentrations (b) from isolated perfused mouse hearts following treatment with AAV9-CMV-SLC25A44 (n=7; purple) versus AAV9-CMV-GFP (n=7; navy). Data represent mean ± SEM.
