# Supplementary material for: Branched-chain α-ketoacids are preferentially reaminated and activate protein synthesis in the heart
Source: Nat Commun. 2021 Mar 15;12:1680. doi: 10.1038/s41467-021-21962-2 (PMC7960706; doi:10.1038/s41467-021-21962-2)
Supplement: Supplementary file 2 — Description of Additional Supplementary Files [file 41467_2021_21962_MOESM2_ESM.docx]

File Name: Supplementary Data 1

Description: Phosphopeptide and protein expression from phosphoproteomics study of hearts perfused with low and high BCKA concentrations.

File Name: Supplementary Data 2

Description: Significantly upregulated (N=28) and downregulated (N=111) phosphopeptides from phosphoproteomics study of hearts perfused with low and high BCKA concentrations. A threshold of 2-fold change and *P*-value <0.01 was used to define significance.

File Name: Supplementary Data 3

Description: Gene ontology (GO) enrichment analysis of upregulated and downregulated phosphoproteins associated with exposure to high BCKA concentrations.
