# Supplementary material for: Branched-chain α-ketoacids are preferentially reaminated and activate protein synthesis in the heart
Source: Nat Commun. 2021 Mar 15;12:1680. doi: 10.1038/s41467-021-21962-2 (PMC7960706; doi:10.1038/s41467-021-21962-2)
Supplement: Supplementary file 6 — Reporting Summary [file 41467_2021_21962_MOESM6_ESM.pdf]

nature research

Corresponding author(s): Robert McGarrah, Phillip White

Last updated by author(s): Jan 26, 2021

Reporting Summary

Nature Research wishes to improve the reproducibility of the work that we publish. This form provides structure for consistency and transparency in reporting. For further information on Nature Research policies, see our Editorial Policies and the Editorial Policy Checklist.

Statistics

For all statistical analyses, confirm that the following items are present in the figure legend, table legend, main text, or Methods section.

n/a	Confirmed	
☐	☒	The exact sample size (n) for each experimental group/condition, given as a discrete number and unit of measurement
☐	☒	A statement on whether measurements were taken from distinct samples or whether the same sample was measured repeatedly
☐	☒	The statistical test(s) used AND whether they are one- or two-sided<br>*Only common tests should be described solely by name; describe more complex techniques in the Methods section.*
☒	☐	A description of all covariates tested
☒	☐	A description of any assumptions or corrections, such as tests of normality and adjustment for multiple comparisons
☐	☒	A full description of the statistical parameters including central tendency (e.g. means) or other basic estimates (e.g. regression coefficient) AND variation (e.g. standard deviation) or associated estimates of uncertainty (e.g. confidence intervals)
☐	☒	For null hypothesis testing, the test statistic (e.g. F, t, r) with confidence intervals, effect sizes, degrees of freedom and P value noted<br>*Give P values as exact values whenever suitable.*
☒	☐	For Bayesian analysis, information on the choice of priors and Markov chain Monte Carlo settings
☒	☐	For hierarchical and complex designs, identification of the appropriate level for tests and full reporting of outcomes
☐	☒	Estimates of effect sizes (e.g. Cohen's d, Pearson's r), indicating how they were calculated

Our web collection on statistics for biologists contains articles on many of the points above.

Software and code

Policy information about availability of computer code

Data collection	Image Studio was used for western blot scanning and signal quantification.<br>QuantStudio 6 Flex Real-Time PCR system was used for qPCR data collection.<br>Thermo Proteome Discoverer 2.4. Database was used for proteomic data analysis.
Data analysis	R version 4.0.2 was used for one-way ANOVAs and Spearman correlations.<br>Excel was used for Student's t-test.<br>STRING v11 was used for generating protein-protein interation networks.

For manuscripts utilizing custom algorithms or software that are central to the research but not yet described in published literature, software must be made available to editors and reviewers. We strongly encourage code deposition in a community repository (e.g. GitHub). See the Nature Research guidelines for submitting code & software for further information.

Data

Policy information about availability of data

All manuscripts must include a data availability statement. This statement should provide the following information, where applicable:

- Accession codes, unique identifiers, or web links for publicly available datasets
- A list of figures that have associated raw data
- A description of any restrictions on data availability

Raw (phospho)proteomics datasets generated during and/or analysed during the current study are available in the MassIVE database, ftp://MSV000086199@massive.ucsd.edu. Metabolomics data is available at the NIH Common Fund's National Metabolomics Data Repository (NMDR) website, the Metabolomics Workbench, https://www.metabolomicsworkbench.org where it has been assigned Project ID (PR001070). Additional datasets generated during and/or analysed during the current study are available from the corresponding author on reasonable request.

Field-specific reporting

Please select the one below that is the best fit for your research. If you are not sure, read the appropriate sections before making your selection.

- [x] Life sciences
- [ ] Behavioural & social sciences
- [ ] Ecological, evolutionary & environmental sciences

For a reference copy of the document with all sections, see nature.com/documents/nr-reporting-summary-flat.pdf

Life sciences study design

All studies must disclose on these points even when the disclosure is negative.

Sample size	Based on our prior experiments using stable-isotope metabolite tracing for in vivo studies and isolated heart perfusions, we have found that a minimum number of four animals per group is needed to detect at least a 20% difference in isotope enrichments across most of our measured metabolites (DOI: 10.1152/ajpendo.00334.2019; DOI: 10.1152/ajpendo.00081.2018; DOI: 10.1016/j.celrep.2020.108375).
Data exclusions	No samples were excluded from the statistical analysis unless specifically stated otherwise. One gastrocnemius specimen was not included for correlation analyses due to missing metabolomics data (Figure 3A). A liver specimen from one animal was not included due to insufficient tissue amounts (Figure 3A).
Replication	The main finding of reverse transamination of BCKA to BCAA in the heart were verified with two different substrates and in two different rodent species. Our ex vivo tracing studies were also validated in an in vivo cohort. For molecular experiments, each single measurement was performed in duplicate and the results were consistently reproducible.
Randomization	For rat studies, each cage (n=2 rats) was randomly assigned to a treatment group (Vehicle, BT2, or LY when applicable). For mouse studies, each cage (n=4 mice) was randomly assigned to AAV9-CMV-GFP or AAV9-CMV-SLC25A44 treatment. Perfusion studies were conducted so multiple samples from each group were run each day.
Blinding	Samples were blinded prior to GC-MS and metabolomic analysis. However, all other studies were not blinded because treatment was administered by the researcher collecting the data.

Reporting for specific materials, systems and methods

We require information from authors about some types of materials, experimental systems and methods used in many studies. Here, indicate whether each material, system or method listed is relevant to your study. If you are not sure if a list item applies to your research, read the appropriate section before selecting a response.

Materials & experimental systems

n/a	Involved in the study	
[ ]	[x]	Antibodies
[ ]	[x]	Eukaryotic cell lines
[x]	[ ]	Palaeontology and archaeology
[ ]	[x]	Animals and other organisms
[x]	[ ]	Human research participants
[x]	[ ]	Clinical data
[x]	[ ]	Dual use research of concern

Methods

n/a	Involved in the study	
[x]	[ ]	ChIP-seq
[x]	[ ]	Flow cytometry
[x]	[ ]	MRI-based neuroimaging

Antibodies

Antibodies used	1. S6 (Cell Signaling Technologies #2317)<br>2. Phospho-S6 (Ser 235/236) (Cell Signaling Technologies #4858)<br>3. 4E-BP1 (Cell Signaling Technologies #9644)<br>4. Phospho-4E-BP1 (Thr 37/46) (Cell Signaling Technologies #2855)<br>5. Pan-actin (Cell Signaling Technologies #8456)<br>6. Puromycin (EMD Millipore #MABE343)<br>7. Beta-tubulin (Sigma Aldrich, T8328)<br>8. Alexa-Fluor Plus 800 (Thermo Fisher, A32735)<br>9. Alexa-Fluor Plus 594 (Thermo Fisher, A32742)
Validation	All antibodies have been validated for immunoblotting applications and validation information can be found on the manufacturer's website. For S6 see https://www.cellsignal.com/products/primary-antibodies/s6-ribosomal-protein-54d2-mouse-mab/2317, phospho-S6 see https://www.cellsignal.com/products/primary-antibodies/phospho-s6-ribosomal-protein-ser235-236-d57-2-2e-xp-rabbit-mab/4858, 4E-BP1 see https://www.cellsignal.com/products/primary-antibodies/4e-bp1-53h11-rabbit-mab/9644, phospho-4E-BP1 see https://www.cellsignal.com/products/primary-antibodies/phospho-4e-bp1-thr37-46-236b4-rabbit-mab/285,

pan-actin see https://www.cellsignal.com/products/primary-antibodies/phospho-4e-bp1-thr37-46-236b4-rabbit-mab/2855, puromycin see https://www.emdmillipore.com/US/en/product/Anti-Puromycin-Antibody-clone-12D10,MM_NF-MABE343, beta-tubulin see https://www.sigmaaldrich.com/catalog/product/sigma/t8328?lang=en&region=US, Alexa-Fluor Plus 800 see https://www.thermofisher.com/antibody/product/Goat-anti-Rabbit-IgG-H-L-Highly-Cross-Adsorbed-Secondary-Antibody-Polyclonal/A32735, and Alexa-Fluor Plus 594 see https://www.thermofisher.com/antibody/product/Goat-anti-Mouse-IgG-H-L-Highly-Cross-Adsorbed-Secondary-Antibody-Polyclonal/A32742.

Eukaryotic cell lines

Policy information about cell lines

Cell line source(s)	HEK293, L6
Authentication	None of the cell lines used were authenticated.
Mycoplasma contamination	The cell lines were not tested for mycoplasma.
Commonly misidentified lines (See ICLAC register)	There were no commonly misidentified cell lines were used.

Animals and other organisms

Policy information about studies involving animals; ARRIVE guidelines recommended for reporting animal research

Laboratory animals	Ten-week old, male, Wistar rats (weight range 250-274 g) were used for the isolated heart perfusion and in vivo studies and were purchased from Envigo. All mice used in this study were 8-week old, male, C57BL/6J mice purchased from Jackson Laboratories. Animals were dual housed in a 12 hour light:dark cycle and given ad-libitum access to a standard chow diet (TD.7001, Harlan Teklad) and water.
Wild animals	No wild animals were used in this study.
Field-collected samples	No field-collected samples were used in this study.
Ethics oversight	Duke University Institutional Animal Care and Use Committee

Note that full information on the approval of the study protocol must also be provided in the manuscript.
